# Improved Process for the Synthesis of 3-(3-Trifluoromethylphenyl)propanal for More Sustainable Production of Cinacalcet HCl †

**DOI:** 10.3390/molecules28166042

**Published:** 2023-08-13

**Authors:** Vikas Damu Rathod, Stefano Paganelli, Marijan Kočevar, Marko Krivec, Oreste Piccolo

**Affiliations:** 1 Dipartimento di Scienze Molecolari e Nanosistemi, Università Ca’ Foscari Venezia, Via Torino 155, 30170 Venezia Mestre, Italy; vikasrathod@iolcp.com (V.D.R.); spag@unive.it (S.P.); 2 Faculty of Chemistry and Chemical Technology, University of Ljubljana, Večna pot 113, SI-1000 Ljubljana, Slovenia; marijan.kocevar@fkkt.uni-lj.si (M.K.); marko.krivec@fkkt.uni-lj.si (M.K.); 3 SCSOP, Via Bornò 5, 23896 Sirtori, Italy

**Keywords:** Pd catalyst, cascade process, C-C and C-H bond formation, microwaves, selective ester reduction

## Abstract

Cinacalcet (**I**), sold as hydrochloride salt, is a calcimimetic drug which has been approved for the treatment of secondary hyperparathyroidism in patients with chronic renal disease and for the treatment of hypercalcemia in patients with parathyroid carcinoma. Here, an improved method for the synthesis of 3-(3-trifluoromethylphenyl)propanal (**II**), a key intermediate for the preparation of **I**, is described. The protocol required a Mizoroki–Heck cross-coupling reaction between 1-bromo-3-(trifluoromethyl)benzene and acroleine diethyl acetal, catalyzed by Pd(OAc)_2_ in the presence of nBu_4_NOAc (tetrabutylammonium acetate), followed by the hydrogenation reaction of the crude mixture of products in a cascade process. Palladium species, at the end of the reaction, were efficiently recovered as Pd/Al_2_O_3_. The procedure was developed under conventional heating conditions as well as under microwave-assisted conditions. The obtained mixture of 1-(3,3-diethoxypropyl)-3-(trifluoromethyl)benzene (**III**), impure for ethyl 3-(3-trifluoromethylphenyl) propanoate (**IV**), was finally treated, under mild conditions, with potassium diisobutyl-*tert*-butoxyaluminum hydride (PDBBA) to obtain after hydrolysis 3-(3-trifluoromethylphenyl)propanal (**II**), in an excellent overall yield and very high purity. Microwave conditions permitted a reduction in reaction times without affecting selectivity and yield. The final API was obtained through reductive amination of (**II**) with (*R*)-(+)-1-(1-naphthyl)ethylamine (**V**) using a catalyst prepared by us with a very low content of precious metal.

## 1. Introduction

**Figure 1:**
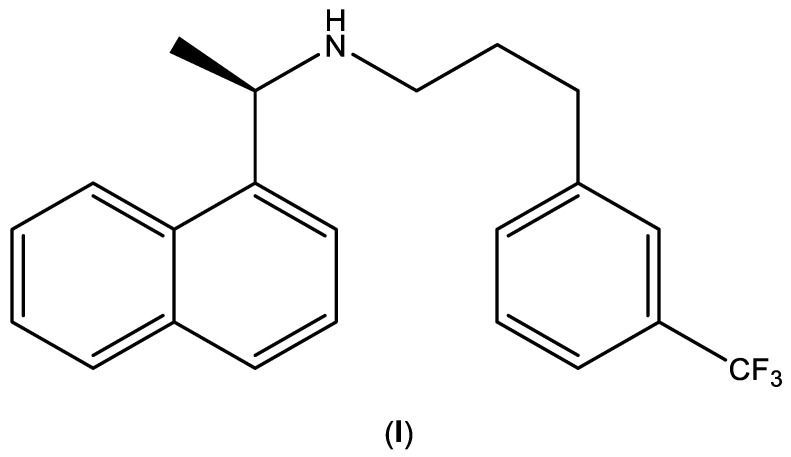
Structure of Cinacalcet (**I**).

The API, Cinacalcet, N-[(*R*)-1-(1-naphthyl)ethyl]-3-[3-(trifluoromethyl)phenyl] propan-1-amine (**I**), (Figure 1), sold as hydrochloride salt (Sensipar^®^ in North America and Australia, Mimpara^®^ in Europe and Regpara^®^ in Asia), is a drug that acts as a calcimimetic, i.e., it mimics the action of calcium on tissues by allosteric activation of the calcium-sensing receptor that is expressed in various human organ tissues.

Its worldwide production in 2022 was about 5.4 tons with a sales value of USD 263 million [1]. The originator patent [2] expired in 2018 in the USA and in 2019 in Europe. Cinacalcet hydrochloride is used to treat secondary hyperparathyroidism (elevated parathyroid hormone levels), a consequence of end-stage renal disease; it is also indicated for the treatment of hypercalcemia in patients with parathyroid carcinoma and for the treatment of secondary hyperparathyroidism in people with chronic kidney disease on dialysis. It can also be used to treat severe hypercalcemia in patients with primary hyperparathyroidism who are unable to undergo parathyroidectomy [3,4].

Given that cinacalcet is a first-in-class drug, several academic groups and generic drug manufacturers have searched for alternative synthetic and more sustainable procedures. A review of many synthetic approaches was published a few years ago [5]; subsequent selected publications are also reported [6,7].

In most processes used to obtain **I**, (*R*)-(+)-1-(1-naphthyl)ethylamine (**V**) is used as the source of chirality, as this enantiopure amine is readily accessible through classical or enzymatic resolution of the racemic precursor [8,9], even if more recently alternative methods have also been described [10,11,12]. This amine **V** is coupled to the other part of the molecule in three main ways: (a) a reaction with an aldehyde followed by reduction of the resulting imine; (b) formation of an amide followed by reduction; or (c) nucleophilic substitution with a suitably substituted partner that carries a leaving group. 

**Scheme 1:**
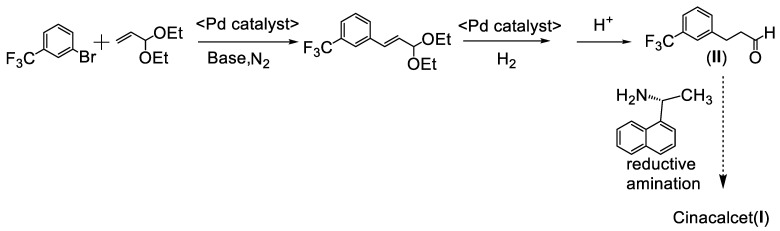
Reaction scheme for the synthesis of Cinacalcet (**I**).

Our research aimed to investigate the total synthesis of Cinacalcet hydrochloride, trying to find greener and sustainable conditions potentially suitable for scaling-up and industrialization. In particular, we investigated alternative methods to obtain the key intermediate **II**. The initial project idea is depicted in Scheme 1.

## 2. Mizoroki–Heck Cross-Coupling Reaction between 1-Bromo-3-(trifluoromethyl)benzene and Acroleine Diethyl Acetal

**Scheme 2:**
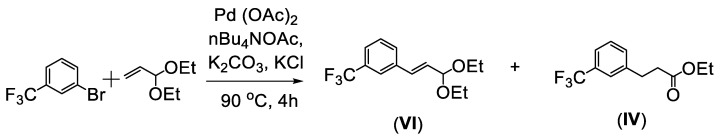
Mizoroki–Heck cross-coupling reaction.

In the first step (Scheme 2), it was decided we would avoid the use of a phosphine ligand [13] reported for this process, and the Mizoroki–Heck cross-coupling reaction was initially studied by utilizing the conditions reported in the literature [14] for the synthesis of the analogous cinnamaldehyde derivatives palladium (**II**) acetate, nBu_4_NOAc, K_2_CO_3_ and KCl in *N*,*N*-dimethylformamide (DMF) at 90 °C for 3–4 h. After the conversion was complete, a mixture of compound 1-((*E*)-3,3-diethoxyprop-1-enyl)-3-(trifluoromethyl) benzene (**VI**) and ethyl 3-(3-(trifluoromethyl)phenyl)propanoate (**IV**) in the ratio 85:15 was obtained.

This encouraging result presented, however, a critical problem, i.e., the use of DMF as a solvent. It is not a green solvent, has toxic reproduction-related effects, according to the European Chemicals Agency (ECHA), is thought to cause birth defects and has been linked to cancer in humans by the International Agency for Research on Cancer (IARC) [15]. Most manufacturers of DMF list it as a ‘life’ or ‘chronic’ health hazard in their MSDS since DMF is not readily disposed by the body. There is also a concrete risk that in the near future, its use will be banned in Europe. For this reason, a detailed study of this reaction was carried out, exploring greener solvents and different reaction parameters to improve the selectivity. In all experiments, palladium acetate was the catalyst. The results are reported in Table 1.

**Scheme 3:**
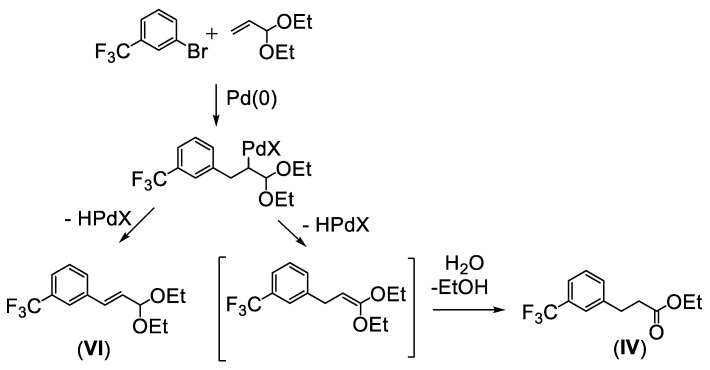
Hypothesized reaction mechanism.

According to [14], a possible mechanism to explain the formation of the two products, 1-((*E*)-3,3-diethoxyprop-1-enyl)-3-(trifluoromethyl)benzene (**VI**) and ethyl 3-(3-(trifluoromethyl)phenyl) propanoate (**IV**), is presented in Scheme 3.

It is reasonable to think that the nature of the base may affect the fate of decomposition of the alkyl palladium intermediate. Actually, a big effect on the selectivity was found when working with different organic and inorganic bases, but, at present, it is difficult to give a clear explanation for this result. If we compare runs 8, 11 and 12 in Table 1, we can suppose that steric hindrance plays an important role, favoring the decomposition of the alkyl palladium intermediate into products. Also, the nature of the X group (halide or acetate) bonded to the palladium intermediate could be important; it underlines the strong effect on selectivity induced when using, as a ligand, nBu_4_NOAc or nBu_4_NBr (run 1 vs. run 6, where the selectivity ratio is 85/15 and 40/60, respectively). Finally, as it is a concerning solvent, it was possible to find two greener solvents that, under the same reaction conditions, led to better selectivity than DMF: γ-valerolactone (Table 1 run 3; 95/5) and 2-Me-THF (Table 1 run 4; 93/7). On the contrary, CPME afforded a less satisfactory result (Table 1 run 5; 80/20). Finally, the best selectivity (Table 1 run 13 vs. run 1) was obtained using nBu_4_NOAc 97% instead of nBu_4_NOAc 90% in DMF. Unfortunately, nBu_4_NOAc 97% is very expensive and is difficult to find in large amounts; for this reason, we studied the reaction using nBu_4_NOAc 90%, which is less expensive and easily available in an industrial amount accepting slightly lower selectivity. Our results for conversion and selectivity are better than those reported in [16].

## 3. Hydrogenation of VI, in a Mixture with IV, by Utilizing the Same Mizoroki–Heck Catalyst Followed by Hydrolysis of 1-(3,3-Diethoxypropyl)-3-(trifluoromethyl)benzene (III) to Afford II in a Mixture with IV

**Scheme 4:**
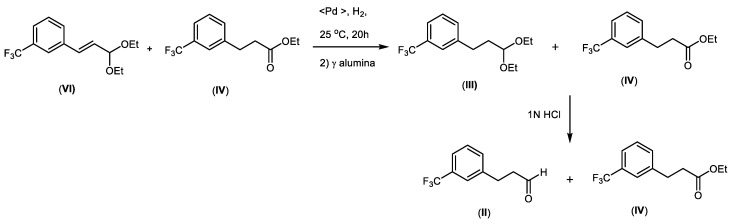
Hydrogenation of the mixture **VI** + **IV** and hydrolysis to afford the mixture **II** + **IV**.

As shown in Scheme 4, it was possible to realize a cascade process, combining the Heck cross-coupling step with the subsequent carbon–carbon double bond hydrogenation^,^ using the same catalyst by simply changing the reaction atmosphere from nitrogen to hydrogen. Under standard optimized conditions (0.1 MPa of H_2_, 25 °C, 20 h in γ-valerolactone or in Me-THF), hydrogenation of **VI** to **III** resulted in a 100% yield as compound **IV** remained unchanged in the reaction mixture. During this synthesis, which has already been described in DMF but without any experimental detail [17], our innovative idea was to recover the palladium species, at the end of the reaction, via absorption on γ-alumina. To the crude hydrogenation reaction mixture alumina was therefore added, and then the suspension was filtered on sintered glass, washed with the used organic reaction solvent to remove the adsorbed product and finally with water to remove soluble inorganic material. After drying the alumina under vacuum, the material was analyzed, showing a palladium content of 0.18%. This recovered catalyst (Pd/Al_2_O_3_) was later used in the reductive amination step. The organic phase was treated with aqueous HCl to cleavage acetal **III** to obtain compound **II** in an excellent yield, >90%, (Scheme 4). The standard reaction conditions were 30 °C for 1–6 h. Longer reaction times were necessary when ethereal solvents, such as Me-THF and CPME, were used, due to the initial inhomogeneity of the reaction mixture. On the contrary, the presence of γ-valerolactone strongly accelerated acetal deprotection. After a work-up and evaporation of the solvent, **II** and the unchanged by-product **IV** were recovered in a quantitative yield.

## 4. Purification of 3-(3-(Trifluoromethyl)phenyl)propanal (II) via Bisulfite Adduct and Regeneration of the Aldehyde

**Scheme 5:**
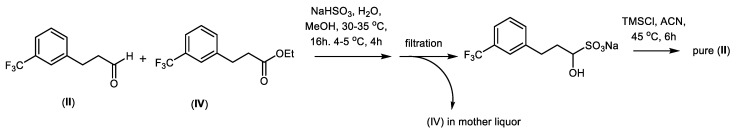
Purification of **II** via Bertagnini adduct.

Purification of crude carbonyl compounds via crystallization of their bisulfite adducts and subsequent regeneration of pure carbonyl derivatives is a known technique [18]. However, it is necessary to tune the reaction conditions to achieve good results, in particular with aldehydes. The crude mixture containing **II**/**IV** (ratio 80–95%/20–5%) quickly gave a precipitate when treated with sodium bisulfite in ethanol/water (2/1); the resulting suspension was stirred at 35 °C for 16 h and subsequently at 5 °C for 4 h; then, the solid was filtered and washed with methyl tert-butyl ether to remove **IV**. The Bertagnini adduct was recovered with a nearly quantitative yield in agreement with the literature [19,20] (Scheme 5).

The regeneration of aldehyde **II** from the Bertagnini adduct was carried out using a method adapted from a literature protocol [21]. Sodium 1-hydroxy-3-(3-trifluoromethylphenyl)propane-1-sulfonate was treated with TMS-Cl at 45 °C, using acetonitrile as a preferred solvent, and the conversion was monitored through ^1^H-NMR analysis of filtered samples. The quantitative transformation into **II** was obtained in about 6 h and after a work-up, highly pure **II** was recovered in a 95% yield.

## 5. Reduction of Ethyl 3-(3-(Trifluoromethyl)phenyl)propanoate by-Product (IV) to 3-(3-(Trifluoromethyl)phenyl)propanal (II)

**Scheme 6:**
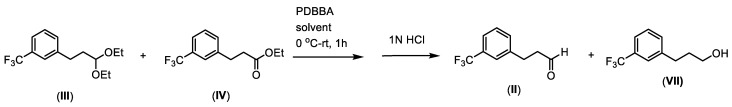
Reduction of **IV** to **II**.

During the present research work, it was decided we would also explore the reduction of the mixture of **III** and **IV**, previously recovered after the hydrogenation step, by potassium diisobutyl-*tert*-butoxy aluminum hydride (PDBBA) [22] (Scheme 6) as well as sodium diisobutyl-*tert*-butoxy aluminum hydride (SDBBA). Diisobutylaluminum hydride (DIBAL-H) was used for a comparison. From the point of view of process optimization to obtain a better yield of the desired product, it was necessary to selectively reduce the by-product **IV** to the desired compound **II**. The results are reported in Table 2.

After an acidic work-up and removing the solvent under vacuum, under the best experimental conditions, the residue contained 97% of **II** and 3% of the by-product **VII**, in a >90% isolated yield. When 1.1 equivalent of DIBAL-H was used, the selectivity was very poor despite the very low reaction temperature (−75 °C) (Table 2, run 7), which was not suitable for industrial scale. No attempt to improve the selectivity with this latter reagent was performed by changing the solvent, because the reaction result was excellent with PDBBA. 

**Scheme 7:**
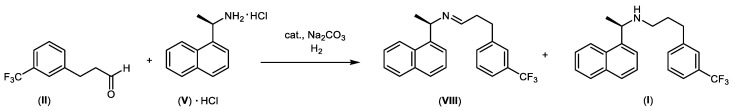
Reductive amination of **II**.

3-(3-(Trifluoromethyl)phenyl)propanal (**II**), obtained via the reactions described in paragraphs 4 or 5, was finally converted into Cinacalcet via reductive amination (Scheme 7), employing homemade low-metal-content catalysts, 0.28% Pd/Al_2_O_3_ and 0.18% Rh/Al_2_O_3_ [23,24], as well as 0.18% Pd/Al_2_O_3_, recovered as described in paragraph 3. The results are reported in Table 3.

It is interesting to note that with these catalysts, neither the formation of alcohol derived from the hydrogenation of **II** nor partial hydrogenation of the naphthalene ring was observed. The isolated yield of pure **I**, under the best reaction conditions, was 94%.

Recently, an improved alternative protocol of reduction amination, avoiding the use of hydrogen and employing NaBH_4_ in the presence of the recyclable heterogeneous greener Lewis acid Aquivion^®^-Fe, has been described by some researchers [25]. This procedure is very suitable when hydrogen is not available or it is preferable not to use it. 

## 6. Synthesis of Cinacalcet Hydrochloride under Microwave Conditions

The advantage of microwaves, as an efficient heating source for organic transformation, was recognized in the mid-1980s. Since then, many successful reactions with dramatically enhanced reaction rates have been disclosed. Very good yields and clean reactions have been obtained using small amounts of energy and short reaction times, and a possible scaling up of the process was discussed [26,27,28,29,30,31]. In this research, to verify the possibility of improving conventional batch results, homogeneous palladium-catalyzed Mizoroki–Heck cross-coupling in a CEM Discover microwave processor equipped with a 300 W power source was studied. The following hydrogenation reaction as well as heterogeneous metal-catalyzed reductive amination reaction was investigated in a CEM Discover single-mode microwave reactor equipped with a 300 W power source and 300 psi pressure source. The best results are reported in Table 4, Table 5 and Table 6.

Under the above-reported conditions, the reaction was completed with a very high yield and in a very short amount of time (see Table 5), but we need to underline that an experiment on a 2 mmol scale was also attempted under the same experimental conditions using 4 mL of 2Me-THF in a 10 mL CEM Discover vial, and this reaction mixture was difficult to hydrogenate under microwave conditions. According to the literature [30], the stirring speed and dilution of the reaction mixture may play a critical role in the outcome of the MW hydrogenation processes. In this experiment, we also made the same observation, obtaining only 40% conversion of compound **VI** to **III**. This is probably due to the fact that the hydrogen contained in the top part of the reaction vial (headspace) needs to diffuse to the liquid phase in which the catalytic process occurs and hydrogen is consumed. Increased stirring influences the gas–liquid interfacial area and thus affects the hydrogenation rate, which is a known phenomenon in hydrogenation chemistry. From a chemical engineering standpoint, it is clear that the 10 mL cylindrical vial used for most microwave experiments in single-mode reactors today, in combination with a comparatively ineffective magnetic stirring system, is not an ideal reactor for gaseous transformations of this type of reaction. Therefore, laboratory MW equipment in batch mode does not lead to great advantages over the conventional batch reaction protocol even if it is possible to considerably reduce the reaction time. This problem should be considered in a future scaled-up investigation.

Working under the conditions reported in Table 6, a 99% yield of the desired product **I** was achieved (run 1); noteworthily, a similar result was also obtained by reducing the amount of catalyst (Table 6, run 2). An incomplete reaction with a high amount of intermediate imine **VIII** was surprisingly found using 2-methyltetrahydrofuran as the solvent under microwave irradiation (Table 6, run 3). A very positive result was achieved by using the homemade catalyst, 0.27% Pt/Al_2_O_3_; the conversion was complete with 98% of the desired product **I** (Table 6, run 4). On the contrary, the 1% Pt/C commercial catalyst, which is often industrially used for this process, under the same experimental conditions, gave disappointing results: a total conversion was always obtained, but only 48% of the sought product I was formed (Table 6, run 5).

Both the homemade heterogeneous catalyst 0.28% Pd/Al_2_O_3_ and 0.27% Pt/Al_2_O_3_ were used with success in this hydrogenation process under microwave irradiation. This result is very encouraging for further applications. In future, it will also be necessary to verify the activity and selectivity of recycled catalysts after microwave irradiation to improve product economy and sustainability.

## 7. Experimental Section

### 7.1. Materials and Reagents

3-Bromobenzotrifluoride (99%), acrolein diethyl acetal (96%), palladium (**II**) acetate (98%), tetrabutylammonium acetate (97%), K_2_CO_3_ (certified AC S, granular powder), KCl (certified ACS, granular powder), *N*,*N*-dimethylformamide (99%), γ-valerolactone (99%), 2-methyl-tetrahydrofuran (>99%), cyclopentyl methyl ether (>99.9%), tetrahydrofuran (99%), lithium hydroxide monohydrate (>99%), 1,8-diazabicyclo[5.4.0]undec-7-ene (98%), sodium methoxide (reagent grade 95%), cesium carbonate (99%), triethylamine (Et_3_N, 99%), *N*,*N*-dicyclohexyl methylamine (97%), sodium bisulfite (mixture of NaHSO_3_ and Na_2_S_2_O_5_), methyl-*tert*-butyl ether, methanol (99%), trimethylsilyl chloride (>99%), acetonitrile (99%), toluene (99%), sodium carbonate (>99%) and diisobutylaluminum hydride (1.0 M solution in tetrahydrofuran) were all purchased from Sigma Aldrich (Italy) and used as received. Tetrabutylammonium acetate (technical grade, 90%) was purchased from TCI. Sodium diisobutyl-*tert*-butoxyaluminum hydride (SDBBA) and potassium diisobutyl-*tert*-butoxyaluminum hydride (PDBBA) were prepared according to the literature [22]. The 1% Pt/C catalyst was purchased from Johnson Matthey (London, UK). Chimet SpA (Area Produttiva, Italy) kindly provided us with alumina (type 49). Low-metal-content catalysts on alumina, including that derived from palladium recovered after the cascade process, were prepared following our known protocols [23,24].

The microwave experiments were performed with CEM Discover in single-mode continuous operation, a focused single-mode microwave reactor equipped with a 300 W power source and 2 MPa pressure limit. A 10 mL fiber-optic accessory was equipped with a gas inlet to allow the introduction of hydrogen gas to the reaction vessel. All reactions were performed in a 10 mL CEM microwave reaction vial. The temperature of the contents of the vessel was monitored using a calibrated infrared temperature controller mounted under the reaction vessel or with a fiber-optic probe (when performing hydrogenation experiments). All of the mixtures were stirred with a Teflon-coated magnetic stir bar in the vessel. Temperature, pressure and power profiles were monitored and recorded using commercially available software provided by the manufacturer of the MW unit.

### 7.2. Characterizations and Measurements

Proton and carbon nuclear magnetic resonance (^1^H-NMR and^13^C-NMR) spectra of the reaction mixtures and products were all obtained at 25 °C on a Bruker Avance spectrometer, Italy (300 MHz), using deuterated chloroform or deuterated dimethyl sulfoxide as the solvent. Bruker 400 (400 MHz) at 25 °C with deuterated chloroform or deuterated dimethyl sulfoxide as the solvent was used for ^19^F-NMR spectra. Proton chemical shifts are reported in ppm (δ) relative to internal tetramethylsilane (TMS, δ 0.00). Carbon chemical shifts are reported in ppm (δ) relative to TMS. Fluorochemical shifts are reported in ppm (δ) relative to neat CFCl_3_ (δ 0.00). GC analysis was performed on an Agilent Technologies 6850 series instrument equipped with an HP-5 (Agilent, Santa Clara, CA, USA) capillary column (30 m × 0.32 mm × 0.25 µm film thickness) and a flame ionization detector (FID). The gas chromatograph is interfaced with a computer. The GC parameters were as follows: initial temperature 50 °C; initial time 5 min; heating ramp 10 °C/min; final temperature 280 °C, final time 5 min; injector temperature 280 °C; detector temperature 280 °C; gas carrier flow (N_2_) 2 mL/min; injection volume 0.1 µL. GC-MS analysis was performed on a ThermoFinnigan (Trace CG 2000) instrument equipped with an HP-5 capillary column (30 m × 0.32 mm × 0.25 µm film thickness) and a quadrupole mass spectrometer (ThermoFinnigan Trace MS) interfaced with a computer. The GC parameters were as follows: initial temperature 60 °C; initial time 5 min; heating ramp 10 °C/min; final temperature 280 °C, final time 5 min; injector temperature 280 °C; detector temperature 280 °C; gas carrier flow (N_2_) 0.8 mL/min; injection volume 0.1 µL.

### 7.3. Syntheses Using Conventional Heating

General procedure for the synthesis of the mixture of 1-((*E*)-3,3-diethoxyprop-1-enyl)-3-(trifluoromethyl)benzene (**VI**) and ethyl 3-(3-(trifluoromethyl)phenyl)propanoate (**IV**)

A total of 10 mL of degassed, used solvent (see Table 1 of the text) was introduced in a dry 50 mL three-neck glass-jacketed round-bottom flask equipped with a reflux condenser, thermometer and magnetic stirring bar, under inert atmosphere. Then, 3-bromobenzotrifluoride (1 g, 4.44 mmol), acrolein diethyl acetal (0.867 g, 6.66 mmol), palladium (**II**) acetate (0.29 mg, 0.133 mmol), 90% tetra-butylammonium acetate (2.67 g, 8.88 mmol), potassium chloride (0.331 g, 4.44 mmol) and potassium carbonate (0.919 g, 3.33 mmol) were added, and the mixture was stirred at 90 °C for 4 h. The reaction was monitored via GC and stopped after the complete conversion of 3-bromobenzotrifluoride. A mixture of the products **VI** and **IV** was obtained. A sample of this mixture was cooled at room temperature and filtered on diatomaceous earth. The upper cake was washed with diethyl ether (20 mL), and the filtrate was washed with water (2 × 10 mL). The organic phase was dried over anhydrous Na_2_SO_4_ and filtered, and the solvent was evaporated under reduced pressure to give a crude mixture of **VI** and **IV** as a yellowish oil. A sample was purified for analytical characterization.

**1-((*E*)-3,3-Diethoxyprop-1-enyl)-3-(trifluoromethyl)benzene (VI):** ^1^H-NMR (300 MHz, CDCl_3_) (Figure S1): 7.66–7.44 (m, 4H), 6.79–6.74 (d, *J* = 15.9 Hz, 1H), 6.33–6.26 (dd, *J* = 16.2, 4.8 Hz, 1H), 5.11–5.09 (dd, *J* = 4.8, 1.2 Hz, 1H), 3.76–3.70 (q, *J* = 6.9, 9.3 Hz), 3.61–3.56 (q, *J* = 7.2, 9.6 Hz, 2H), 1.30–1.25 (t, *J* = 14.1 Hz, 6H); ^13^C-NMR (76 MHz, CDCl_3_) (Figure S2): 144.80, 134.83, 131.35, 129.97, 129.61, 129.53, 128.90, 127.55, 125.15, 119.36, 60.69, 14.15; GC-MS *m*/*z*: 274 [M]^+^; 255 [M, -F]^+^; 229 [M, -C_2_H_5_O]^+^; 200 [M, -C_4_H_10_O]^+^; 172 [M, -C_5_H_10_O_2_ ]^+^; 103 [M, -C_6_H_10_F_3_O_2_]^+^.

We did not find NMR data for **VI** in the literature.

**Ethyl 3-(3-(trifluoromethyl)phenyl)propanoate (IV)**: ^1^H-NMR (300 MHz, CDCl_3_) (Figure S3): 7.47–7.37 (m, 4H), 4.18–4.07 (q, *J* = 16.2, 7.2 Hz, 2H), 3.05–3.00 (t, *J* = 16.5, 8.7 Hz, 2H), 2.68–2.62 (t, *J* = 16.8, 7.5 Hz, 2H), 1.26–1.21 (t, *J* = 9.3, 6.9 Hz, 3H); ^13^C-NMR (50 MHz, CDCl_3_) (Figure S4): 172.06, 141.20, 131.47, 128.57, 124.61, 122.69, 60.18, 35.21, 13.69; GC-MS *m*/*z*: 246 [M]^+^; 227 [M, -F]^+^; 201 [M, -C_2_H_5_O]^+^; 172 [M, -C_3_H_6_O_2_]^+^; 172 [M, -C_5_H_10_O_2_]^+^; 103 [M, -C_6_H_10_F_3_O_2_]^+^.

The ^1^H-NMR and ^13^C-NMR data agree with the reported data [31].

General procedure for the synthesis of the mixture of 1-(3,3-diethoxypropyl)-3-(trifluoromethyl)benzene (**III**) and ethyl 3-(3-(trifluoromethyl)phenyl)propanoate (**IV**)

A total of 10 mL of degassed, used solvent (see paragraph 3) was introduced in a dry 50 mL three-neck glass-jacketed round-bottom flask equipped with a reflux condenser, thermometer and magnetic stirring bar, under inert atmosphere. Then, 3-bromobenzotrifluoride (1 g, 4.44 mmol), acrolein diethyl acetal (0.867 g, 6.66 mmol), palladium acetate (0.29 mg, 0.133 mmol), 90% tetra-butylammonium acetate (2.67 g, 8.88 mmol), potassium chloride (0.331 g, 4.44 mmol) and potassium carbonate (0.919 g, 3.33 mmol) were added, and the mixture was stirred at 90 °C for 4 h. The reaction was monitored via GC and stopped after the complete conversion of 3-bromobenzotrifluoride. Then, the reaction flask was flushed with H_2_ and stirred with 0.1 MPa H_2_ (with a balloon pressure) for 20 h at 30 °C. The reaction flask was then cooled to room temperature under stirring and 4 g of alumina was added to recover palladium species. Then, the suspension was filtered on sintered glass, and the solid was washed twice with diethyl ether and finally with water. The mother liquor was analyzed via GC. A 100% conversion of substrate **VI** into product **III** was detected, while **IV** remained unreacted in the reaction mixture. The organic phase was separated and dried over anhydrous Na_2_SO_4_ and filtered, and the solvent was evaporated under reduced pressure to afford a mixture of **III** and **IV** as a yellowish liquid with a quantitative yield with respect to compound **VI**. A sample was purified for analytical characterization.

**1-(3,3-Diethoxypropyl)-3-(trifluoromethyl)benzene (III)**: ^1^H-NMR (300 MHz, CDCl_3_) (Figure S5): 7.47–7.37 (m, 4H), 4.52–4.46 (t, *J* = 5.7, 9.9 Hz, 1H), 3.75–3.63 (q, *J* = 7.2, 9.6 Hz, 2H), 3.54–3.43 (q, *J* = 6.9, 9.3 Hz, 2H), 2.81–2.73 (q, *J* = 8.1, 10.2 Hz, 2H), 2.02–1.91 (q, *J* = 8.1, 10.1 Hz, 2H), 1.26–1.22 (t, *J* = 7.2, 11.4 Hz, 6H); ^13^C-NMR (50 MHz, CDCl_3_) (Figure S6): 142.46, 131.57, 128.53, 124.84, 124.76, 122.44, 122.36, 101.67, 60.91, 34.62, 30.43, 14.98; GC-MS *m*/*z*: 276 [M]^+^; 257 [M, -F]^+^; 231 [M, -C_2_H_5_O]^+^; 201 [M, -C_4_H_11_O]^+^; 183 [C_4_H_10_FO]^+^; 103 [M, -C_6_H_12_F_3_O_2_]^+^.

We did not find NMR data for **III** in the literature.

Acid treatment of the mixture of 1-(3,3-diethoxypropyl)-3-(trifluoromethyl)benzene (**III**) and ethyl 3-(3-(trifluoromethyl)phenyl)propanoate (**IV**)

In a dry 25 mL single-neck round-bottom flask, the mixture of 1-(3,3-diethoxypropyl)-3-(trifluoromethyl)benzene (**III**) and ethyl 3-(3-(trifluoromethyl)phenyl)propanoate (**IV**) obtained according to b) was introduced; then, 1 M HCl aqueous solution was added dropwise, maintaining the temperature below 25 °C, till pH = 1–2. Then, the mixture was stirred for 30 min (the reaction was monitored by GC), and after completion, the reaction mixture was diluted with diethyl ether (20 mL) and washed with water (20 mL × 3). The organic solutions were combined, dried over anhydrous Na_2_SO_4_ and filtered, and the solvent was evaporated under reduced pressure to quantitatively give 3-(3-(trifluoromethyl)phenyl) propanal (**II**), impure of **IV**.

Purification of the mixture of 1-(3,3-diethoxypropyl)-3-(trifluoromethyl)benzene (**III**) and ethyl 3-(3-(trifluoromethyl)phenyl)propanoate (**IV**) through Bertagnini salt formation

In a dry 50 mL glass-jacketed three-neck round-bottom flask equipped with a thermometer and magnetic stirrer, under inert atmosphere, the mixture of crude **III** and **IV** (1 g) in 45 mL of ethanol was introduced. The resulting solution was stirred at room temperature for 10 min, and then a solution of NaHSO_3_ (0.515 g) in 1.8 mL of water was added dropwise within 10 min. Then, it was stirred at 35 °C for 16 h and subsequently at 5 °C for 4 h. After completion of the reaction, the product was filtered, washed with methyl-*tert*-butyl ether (10 mL × 3) and dried under vacuum to give pure sodium 1-hydroxy-3-(3-trifluoromethylphenyl)propane-1-sulfonate as a white powder.

**Sodium 1-hydroxy-3-(3-trifluoromethylphenyl)propane-1-sulfonate**: ^1^H-NMR (300 MHz, DMSO-d_6_) (Figure S7): 7.81–7.25 (m, 4H), 5.52–5.50 (d, *J* = 6 Hz, 1H), 3.84–3.78 (m, 1H), 2.94–2.81 (m, 1H), 2.76–2.65 (m, 1H), 2.11–2.00 (m, 1H), 1.85–1.73 (m, 1H); ^13^C-NMR (76 MHz, DMSO-d_6_) (Figure S8): 144.17, 133.10, 129.69, 129.25, 126.63, 125.23, 122.87, 82.14, 34.05, 31.57; ^19^F NMR (400 MHz, DMSO-d_6_) (Figure S9): −60.9 (3F).

The ^1^H-NMR and ^13^C-NMR data agree with the reported data [19]. Other data, using D_2_O as an NMR solvent, are reported in [20].

Procedure for the regeneration of 3-(3-(trifluoromethyl)phenyl)propanal (**II**) from sodium 1-hydroxy-3-(3-trifluoromethylphenyl)propane-1-sulfonate

In a dry two-neck glass-jacketed round-bottom flask equipped with a reflux condenser, thermometer and magnetic stirrer, under inert atmosphere, sodium 1-hydroxy-3-(3-trifluoromethylphenyl)propane-1-sulfonate (1.44 g, 19.3 mmol) was introduced in 15 mL of acetonitrile and TMS-Cl (1.75 g, 16.1 mmol); after the addition, the mixture was stirred at 45 °C for 6 h. Initially, the reaction mixture was likely homogeneous, but after 6 h, precipitation of NaCl was observed. The reaction was monitored via GC and GC-MS. After completion, the reaction mixture was cooled at room temperature, and then the resulting mixture was diluted with toluene (20 mL) and washed with water (10 mL × 3) and brine (10 mL × 3). The organic solutions were combined, dried over anhydrous Na_2_SO_4_ and filtered, and the solvent was evaporated under reduced pressure to half volume to give a colorless liquid. This aldehyde **II** containing toluene was ready to use for the next step. The sample was dried, removing toluene for analytical characterization.

**3-(3-(Trifluoromethyl)phenyl)propanal** (**II**): ^1^H-NMR (300 MHz, CDCl_3_) (Figure S10): 9.75 (s, 1H), 7.38–7.31 (m, 4H), 2.95–2.91 (t, *J* = 7.5, 15 Hz, 2H), 2.77–2.73 (t, *J* = 7.8, 14.9 Hz, 1H), 2.66–2.62 (t, *J* = 7.5, 15, 1H); ^13^C-NMR (76 MHz, CDCl_3_) (Figure S11): 200.67, 141.33, 131.82, 129.03, 125.02, 123.22, 44.94, 27.76; GC-MS *m*/*z*: 202 [M]^+^; 183 [M, -F]^+^; 160 [M, -C_2_H_5_O]^+^; 133 [M, -F_3_]^+^; 183 [C_4_H_10_FO]; 103 [M, -C_6_H_12_F_3_O_2_]^+^.

^1^H-NMR and ^13^C-NMR data agree quite well with the reported data [31,32,33,34]. In the literature, ^19^F NMR (565 MHz, CDCl3) δ –62.7 (3F) is also reported [34].

Procedure for the synthesis of 3-(3-(trifluoromethyl)phenyl)propanal (**II**) via selective reduction with potassium diisobutyl-tert-butoxyaluminum hydride (PDBBA) of the mixture of 1-(3,3-diethoxypropyl)-3-(trifluoromethyl)benzene (**III**) and ethyl 3-(3-(trifluoromethyl)phenyl)propanoate (**IV**)

In a thoroughly oven-dried 10 mL single-neck round-bottom flask equipped with a septum, under inert atmosphere, the mixture of 1-(3,3-diethoxypropyl)-3-(trifluoromethyl)benzene (**III**) and ethyl 3-(3-(trifluoromethyl)phenyl)propanoate (**IV**) (87.5 mg, 0.356 mmol) in anhydrous toluene (5 mL) was introduced. This reaction mixture was cooled to 0 °C, and then a solution of PDBBA (potassium diisobutyl-*tert*-butoxyaluminum hydride) (1.25 molar equivalent to the product **IV**, 0.5 M in THF: hexane) was added dropwise with a syringe; after the addition, the temperature was raised to room temperature and the resulting mixture was stirred for 1 h. The reaction mixture was analyzed via GC and GC-MS, and 97% conversion of **IV** to **II** and 3% alcohol **VII** was obtained. The resulting reaction mixture was hydrolyzed by adding 1 M HCl (5 mL) until pH = 1–2, and the product was extracted in diethyl ether (10 mL × 2). The organic layer was washed with water and brine and the organic phase was separated and dried over anhydrous Na_2_SO_4_, filtered and evaporated under reduced pressure to obtain product **II** (96% total yield).

General procedure for the synthesis of 3-(3-(trifluoromethyl)phenyl)-N-((R)-1-(naphthalen-1-yl)ethyl)propan-1-amine (**I**) in the presence of low-metal-content catalysts

In a Schlenk tube under inert atmosphere, 3-(3-(trifluoromethyl)phenyl)propanal (**II**) (1 g, 4.95 mmol), (*R*)-1-(naphthalen-1-yl)ethanamine (**V**) as hydrochloride salt (1.02 g, 4.95 mmol), sodium carbonate (0.524 g, 4.95 mmol), 0.28% Pd/Al_2_O_3_ (0.188 g, sub/cat molar ratio 1000/1) and 10 mL of toluene were introduced. Then, the Schlenk tube was transferred into a 150 mL stainless-steel autoclave under inert atmosphere, pressurized with 0.5 MPa H_2_ and stirred for 24 h at 50 °C. Then, the reactor was cooled to room temperature and the residual gas released. The catalyst was filtered off by using a sintered-glass filter to recover the catalyst for further use, and the organic solution was analyzed via GC and GC-MS. A 100% conversion of substrate **II** into product **I** (98%) and **VIII** (2%) was detected. Then, the obtained filtrate was diluted with fresh toluene (10 mL); the resulting filtrate was washed with water (10 mL × 2) and brine (10 mL × 2), and the organic solution was dried over Na_2_SO_4_, filtered and concentrated under reduced pressure to give a yellowish oil. This oil was dissolved in *n*-pentane (1.5 mL) and 3-(3-(trifluoromethyl)phenyl)-*N*-((R)-1-(naphthalen-5-yl)ethyl)propan-1-amine (**I**) precipitated as a white solid (94% yield).

**3-(3-(Trifluoromethyl)phenyl)-*N*-((*R*)-1-(naphthalen-1-yl)ethyl)propan-1-amine (I)**: ^1^H-NMR (300 MHz, CDCl_3_) (Figure S12): 8.13–8.11 (d, *J* = 9.3 Hz 1H), 7.82–7.78 (dd, *J* = 7.2, 8.1 Hz, 1H), 7.69–7.67 (d, *J* = 8.1 Hz, 1H), 7.58–7.56 (d, *J* = 6.3 Hz, 1H), 7.44–7.24 (m, 6H), 4.58–4.51 (q, *J* = 6.6 Hz, 1H), 2.75–2.49 (m, 4H), 1.81–1.72 (m, 2H), 1.44–1.42 (d, *J* = 6.6 Hz, 3H); GC-MS *m*/*z*: 357 [M]^+^; 342 [M, -CH_3_]^+^; 155 [M, -C_10_H_11_F_3_N]^+^.

The ^1^H-NMR data agree quite well with the reported data for CDCl_3_ and in DMSO-d6 [31,32,35].

Procedure for the preparation of the hydrochloride salt of 3-(3-(trifluoromethyl)phenyl)-N-((R)-1-(naphthalen-1-yl)ethyl)propan-1-amine (**I**)

In a dry 50 mL two-neck round-bottom flask under inert atmosphere, 3-(3-(trifluoromethyl)phenyl)-*N*-((*R*)-1-(naphthalen-1-yl)ethyl)propan-1-amine (**I**) (1.60 g, 4.48 mmol) was introduced in 15 mL of dry ether HCl. The resulting mixture was stirred at 25 °C for 1–2 h, and the obtained precipitated solid, after filtration on a Buchner funnel, was washed with (10 mL × 3) of *n*-pentane to recover 1.74 g (99% yield) of 3-(3-(trifluoromethyl)phenyl)-*N*-((*R*)-1-(naphthalen-1-yl)ethyl)propan-1-amine hydrochloride as a white-crystal solid.

**3-(3-(trifluoromethyl)phenyl)-*N*-((*R*)-1-(naphthalen-1-yl)ethyl)propan-1-amine hydrochloride**: ^1^H-NMR (300 MHz, CDCl_3_) (Figure S13): 10.48 (broad s, 1H), 9.96 (broad s, 1H), 8.16–8.14 (d, *J* = 9 Hz, 1H), 7.90–7.80 (m, 3H), 7.57–7.46 (m, 3H), 7.26–7.12 (m, 4H), 5.16–5.08 (m, 1H), 2.72–2.64 (m, 2H), 2.46–2.39 (m, 2H), 2.23–2.15 (m, 2H), 1.94–1.86 (d, *J* = 6 Hz 1H); ^13^C-NMR (76 MHz, CDCl_3_) (Figure S14): 140.78, 133.86, 132.09, 131.54, 130.69, 129.57, 129.50, 128.85, 127.35, 126.27, 126.12, 125.00, 124.90, 123.1, 121.25, 53.53, 45.49, 32.50, 27.30, 21.31; ^19^F NMR (400 MHz, CDCl_3_) (Figure S15): −62.6 (3F); GC-MS *m*/*z*: 393 [M]^+^; 378 [M, -CH_3_]^+^; 191 [M, -C_10_H_11_F_3_N]^+^.

The ^1^H-NMR and ^13^C-NMR data agree quite well with the reported data in DMSO-d6 [19,32].

## 8. Conclusions

In this work, we studied an alternative protocol to prepare a 3-(3-trifluoromethylphenyl)propanal (**II**) intermediate for more sustainable production of Cinacalcet (**I**) as well as the synthesis of the final API, using conventional heating conditions and exploring the feasibility of an MW heating system. The more relevant results can be summarized as follows:

1. A cascade reaction was applied for the Mizoroki–Heck cross-coupling and the following hydrogenation step using the same palladium species as a catalyst, simply changing the gas atmosphere and using greener solvents than DMF;
2. Palladium species were also recovered in a simple way at the end of both reactions, being described in point (a) as a heterogeneous catalyst on alumina that has been used in the reductive amination step;
3. Two different protocols were applied to purify compound **II** using a Bertagnini salt approach or selective reduction under very mild conditions of by-product **IV**, which was transformed with a high yield and purity into compound **II**;
4. Reductive amination with recyclable heterogeneous catalysts, prepared using a simple protocol developed by us, was efficiently used;
5. The preparation of the API described in this paper is, in our opinion, very efficient and potentially suitable for industrial production;
6. Preliminary results using MW permitted a reduction in the reaction times of some steps in the synthesis of the API, even if idoneous equipment will be required for future scaled-up investigations.

## Figures and Tables

**Table 1:** Mizoroki–Heck reaction results in different conditions.

Run ^a^	Ligand	Solvent	Base	Additive	Conv. [%]	VI Yield %	IV Yield %
**1**	nBu_4_NOAc	DMF	K_2_CO_3_	KCl	100	85	15
**2**	nBu_4_NOAc	DMF	K_2_CO_3_	-	100	80	20
**3**	nBu_4_NOAc	γ-valerolactone	K_2_CO_3_	KCl	100	95	5
**4**	nBu_4_NOAc	2-Me-THF	K_2_CO_3_	KCl	100	93	7
**5**	nBu_4_NOAc	CPME	K_2_CO_3_	KCl	100	80	20
**6**	nBu_4_NBr	DMF	K_2_CO_3_	KCl	100	40	60
**7**	nBu_4_NBr	DMF	LiOH.H_2_O	KCl	100	60	40
**8**	nBu_4_NBr	DMF	DBU	-	58	58	-
**9**	nBu_4_NBr	DMF	NaOMe	-	1	-	1
**10**	nBu_4_NBr	DMF	Cs_2_CO_3_	-	29	7	22
**11**	nBu_4_NBr	DMF	Et_3_N	-	66	1	65
**12**	nBu_4_NBr	DMF	DCHMA	-	95	50	45
**13 ^b^**	nBu_4_NOAc	DMF	K_2_CO_3_	KCl	100	99	1

Reaction conditions: ^a^ 1-Bromo-3-(trifluoromethyl)-benzene (1 g, 4.44 mmol), 3,3-diethoxyprop-1-ene (0.867 g, 6.66 mmol), Pd(OAc)_2_ (29 mg, 0.133 mmol), nBu_4_NOAc 90% (1.33 g, 8.88 mmol), KCl (0.33 g, 4.44 mmol), K_2_CO_3_ (0.919 g, 6.66 mmol), T = 90 °C, solvent = 10 mL, t = 4 h. ^b^ Same conditions as those used for run 1, but 2.67 g (8.88 mmol) of nBu_4_NOAc 97% was used. Amounts (%) of products were determined via GC and GC-MS using n-dodecane as internal standard.

**Table 2:** Reduction of ethyl 3-(3-(trifluoromethyl)phenyl)propanoate (**IV**) to 3-(3-(trifluoromethyl)phenyl)propanal (**II**).

Run ^a^	Reagent	Molar amount Based to IV	T (°C)/ t (h)	Solvent	Conv. of IV [%]	II [%]	VII [%]
**1**	PDBBA	1.3	0–25/4	Toluene	100	96	4
**2**	PDBBA	1.3	0–25/4	DCM	100	92	8
**3**	PDBBA	1.3	0–25/4	THF	100	90	10
**4 ^b^**	PDBBA	1.25	0–25/1	Toluene	100	97	3
**5**	SDBBA	1.3	0–25/4	Toluene	100	90	10
**6**	SDBBA	1.3	0–25/4	DCM	100	85	15
**7 ^c^**	DIBALH	1.1	−75/1	THF	100	60	40

Reaction conditions: ^a^ mixture of substrates **III** and **IV** (total amount: 0.5 g, of which 87.5 mg (0.355 mmol) is **IV**), PDBBA solution (0.910 mL, 1.3 equiv. referred to **IV**, 0.5 M solution in THF-heptane), T = 0–25 °C, t = 1–4 h, solvent = 5 mL; ^b^ same conditions of run 1, but 1.25 equiv. of PDBBA; ^c^ same conditions as those used for run 1, but 1.1 equiv. of DIBALH was used and T = −75 °C. Amounts (%) of products were determined via GC and GC-MS, using *n*-dodecane as internal standard.

**Table 3:** Reductive amination of 3-(3-(trifluoromethyl)phenyl)propanal (**II**).

Run	Catalyst	Conv. [%]	VIII %	I %
1 ^a^	0.28% Pd/Al_2_O_3_	100	2	98
2 ^b^	0.28% Pd/Al_2_O_3_	100	1	99
3 ^b^	0.28% Pd/Al_2_O_3_	100	1	99
4 ^b^	0.28% Pd/Al_2_O_3_	100	3	97
5 ^c^	0.18% Rh/Al_2_O_3_	100	38	68
6 ^d^	0.18% Pd/Al_2_O_3_	100	3	97
7 ^b,d^	0.18% Pd/Al_2_O_3_	100	3	97
8 ^b,d^	0.18% Pd/Al_2_O_3_	100	3	97
9 ^b,d^	0.18% Pd/Al_2_O_3_	100	4	96
10 ^b,d^	0.18% Pd/Al_2_O_3_	100	4	96

Reaction conditions: ^a^ **II** = (1 g,4.95 mmol), **V** as hydrochloride = (1.02 g, 4.95 mmol), Na_2_CO_3_ (0.524 g, 4.95 mmol), 0.28% Pd/Al_2_O_3_ (0.188 g, **II**/cat. molar ratio 1000/1), p(H_2_) = 0.5 MPa, T = 50 °C, t = 24 h; toluene = 5 mL; ^b^ reaction carried out using the catalyst recovered from the previous run; ^c^ same conditions as those used for run 1, but 0.18% Rh/Al_2_O_3_ (0.283 g, **II**/cat. molar ratio 1000/1) was used; ^d^ same conditions as those used for run 1, but 0.18% Pd/Al_2_O_3_ (0.283 g, **II**/cat. molar ratio 200/1) was used and t = 6 h. Amounts (%) of products were determined via GC and GC-MS, using commercial **I** as reference standard.

**Table 4:** Pd(OAc)_2_-catalyzed Mizoroki–Heck cross-coupling reaction under microwave irradiation.

Run ^a^	Solvent	Conv. (%)	VI (%)	IV (%)
**1**	2-Me-THF	100	92	8
**2**	γ-Valerolactone	100	91	9
**3**	THF	100	90	10

Reaction conditions: ^a^ 1-Bromo-3-(trifluoromethyl)-benzene (0.240 g, 1 mmol), 3,3-diethoxyprop-1-ene: (0.208 g, 1.59 mmol), Pd(OAc)_2_ (7.1 mg, 0.0326 mmol), n-Bu_4_NOAc (90%) (0.714 g, 2.1 mmol), T = 90 °C, solvent = 2 mL. Microwave conditions: power 5 W, ramp time 5 min, hold time 20 min, pressure limit 2 MPa. Amounts (%) of products were determined via GC and GC-MS using n-dodecane as internal standard.

**Table 5:** Hydrogenation of mixture obtained in the previous step under microwave irradiation.

Run ^a^	Conv. (%)	Time (h)	III (%)	IV (%)
1	91	2	91	8
2	100	4	92	8

Reaction conditions: ^a^ mixture of substrates **VI** (92%) and **IV** (8%)] (1 mmol scale), p(H_2_) = 0.2 MPa, T = 40 °C, 2-MeTHF = 2 mL. Amounts (%) of products were determined via GC and GC-MS, using n-dodecane as internal standard.

**Table 6:** Reductive amination of 3-(3-(trifluoromethyl)phenyl)propanal (**II**) with (R)-1-(naphthalen-1-yl)ethanamine (**V**) as hydrochloride salt under microwave irradiation.

Run ^a^	T (°C)/t (h)	Conv.%	VIII%	I%
1	60/8	100	-	100
2 ^b^	60/8	100	1	99
3 ^c^	60/8	100	48	42
4 ^d^	60/8	100	2	98
5 ^e^	60/8	100	52	48

Reaction conditions: ^a^ **II** (100 mg, 0.5 mmol), **V** as hydrochloride salt (102 mg, 0.5 mmol), Na_2_CO_3_ (52 mg, 0.5 mmol), 0,28% Pd/Al_2_O_3_ (35.12 mg, **II**/catalyst molar ratio 500/1), p(H_2_) = 0.5 MPa, toluene = 2 mL. Microwave conditions: power 20 W to reach the initial temperature, then 2 W, ramp time 5 min, hold time until the end of reaction, H_2_ pressure limit 2 Mpa; ^b^ **II**/catalyst molar ratio 1000/1 was used; ^c^ 2-MeTHF was used as solvent; ^d^ same conditions as those used for run 2, but 0.27% Pt/Al_2_O_3_ (35.7 mg) was used; ^e^ same conditions as those used for run 2, but commercial 1% Pt/C (9.6 mg) was used. Amounts (%) of products were determined via GC and GC-MS, using reference standards.

## Data Availability

The data presented in this study are available in the present article and Supplementary Materials.

## Supplementary Materials

The following supporting information can be downloaded at: https://www.mdpi.com/article/10.3390/molecules28166042/s1. The ^1^H-NMR and ^13^C-NMR spectra of the compounds prepared in this work and ^19^F-NMR spectra of sodium 1-hydroxy-3-(3-trifluoromethylphenyl)propane-1-sulfonate and 3-(3-(trifluoromethyl)phenyl)-*N*-((*R*)-1-(naphthalen-1-yl)ethyl)propan-1-amine hydrochloride.
