# Supplementary material for: Improved Process for the Synthesis of 3-(3-Trifluoromethylphenyl)propanal for More Sustainable Production of Cinacalcet HCl
Source: Molecules. 2023 Aug 13;28(16):6042. doi: 10.3390/molecules28166042 (PMC10458415; doi:10.3390/molecules28166042)
Supplement: Supplementary file 1 [file molecules-28-06042-s001.zip › molecules-2509052-supplementary.pdf]

Supplementary Materials

Improved process for the synthesis of 3-(3-trifluoromethylphenyl)propanal for more sustainable production of Cinacalcet HCl[†]

Vikas Damu Rathod 1,‡, Stefano Paganelli 1, Marijan Kočevar 2, Marko Krivec 2 and Oreste Piccolo 3,*

1 Dipartimento di Scienze Molecolari e Nanosistemi, Università Ca' Foscari Venezia, Via Torino 155, 30170 Venezia Mestre, Italy; vikasrathod@iolcp.com (V.D.R.); spag@unive.it (S.P.)

2 Faculty of Chemistry and Chemical Technology, University of Ljubljana, Večna pot 113,

SI-1000 Ljubljana, Slovenia; marijan.kocevar@fkkt.uni-lj.si (M.K.); marko.krivec@fkkt.uni-lj (M.K.)

3 SCSOP, Via Bornò 5, 23896 Sirtori, Italy

* Correspondence: oreste.piccolo@scsop.it; Tel.: +39-3939479870

† This work was a part of the PhD project of VDR performed at University of Venice (batch procedures) and at University of Ljubljana (MW experiments).

‡ Present address: IOL Chemicals and Pharmaceuticals Ltd., 85, Industrial Area A, Ludhiana 141003, Punjab, India.

Spectra data of the products

^{1}H-NMR and ^{13}C-NMR spectra of the compounds prepared in this work are reported here as well as ^{19}F-NMR spectra of Sodium 1-hydroxy-3-(3-trifluoromethylphenyl)propane-1-sulfonate and of 3-(3-(trifluoromethyl)phenyl)-*N*-((*R*)-1-(naphthalen-1-yl)ethyl)propan-1-amine hydrochloride. All compounds are known but for compounds 1-(3,3-Diethoxypropyl)-3-(trifluoromethyl)benzene (**III**) and 1-((*E*)-3,3-Diethoxyprop-1-enyl)-3-(trifluoromethyl)benzene (**VI**), at the best of our knowledge, NMR spectra were not previously reported.

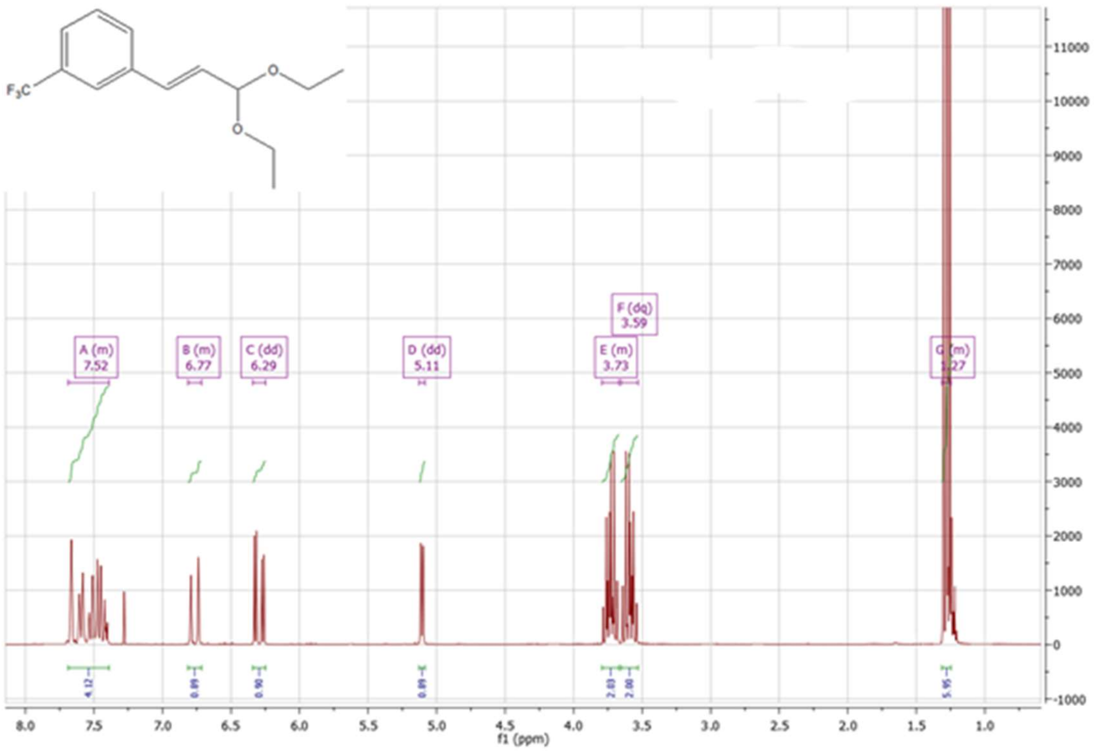

Fig S1. ^{1}H-NMR of 1-((*E*)-3,3-Diethoxyprop-1-enyl)-3-(trifluoromethyl)benzene (VI) (300MHz, $CDCl_3$): 7.66-7.44 (m, 4H), 6.79-6.74 (d, *J* = 15.9 Hz, 1H), 6.33-6.26 (dd, *J* = 16.2, 4.8 Hz, 1H), 5.11-5.09 (dd, *J* = 4.8, 1.2 Hz, 1H), 3.76-3.70 (q, *J* = 6.9, 9.3 Hz), 3.61-3.56 (q, *J*= 7.2, 9.6 Hz, 2H), 1.30-1.25 (t, *J* = 14.1 Hz, 6H).

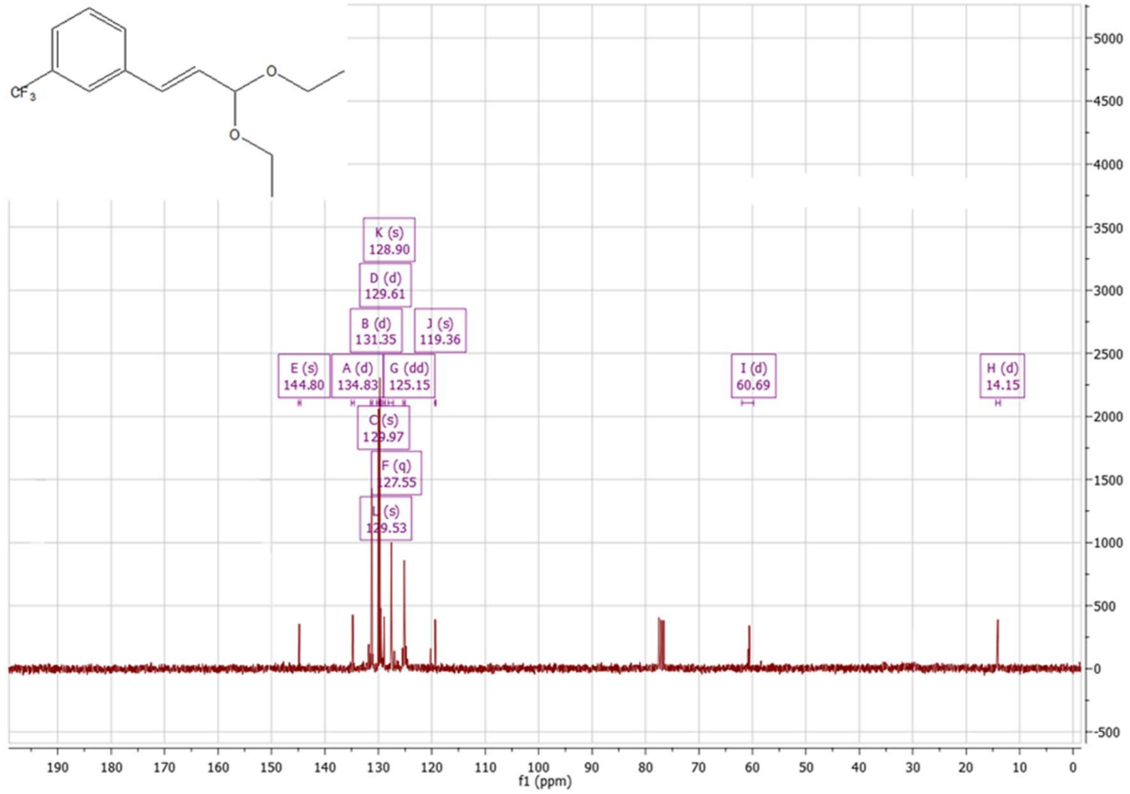

Fig S2. ^{13}C-NMR of 1-((*E*)-3,3-Diethoxyprop-1-enyl)-3-(trifluoromethyl)benzene (VI) (76 MHz, $CDCl_3$) : 144.80, 134.83, 131.35, 129.97, 129.61, 129.53, 128.90, 127.55, 125.15, 119.36, 60.69, 14.15.

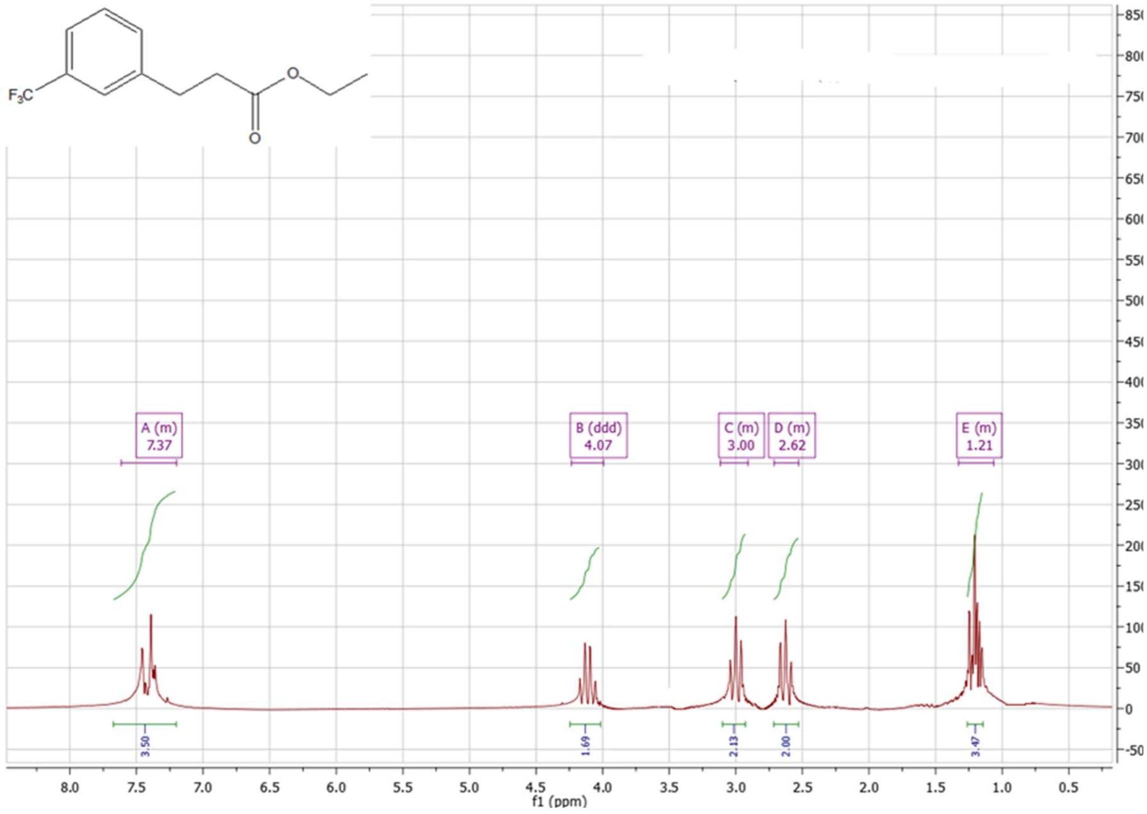

Fig S3. ^{1}H-NMR of Ethyl 3-(3-(trifluoromethyl)phenyl)propanoate (IV) (300MHz, $CDCl_3$): 7.47-7.37 (m, 4H), 4.18-4.07 (q, J = 16.2, 7.2 Hz, 2H), 3.05-3.00 (t, J = 16.5, 8.7 Hz, 2H), 2.68-2.62 (t, J = 16.8, 7.5 Hz, 2H), 1.26-1.21 (t, J = 9.3, 6.9 Hz, 3H).

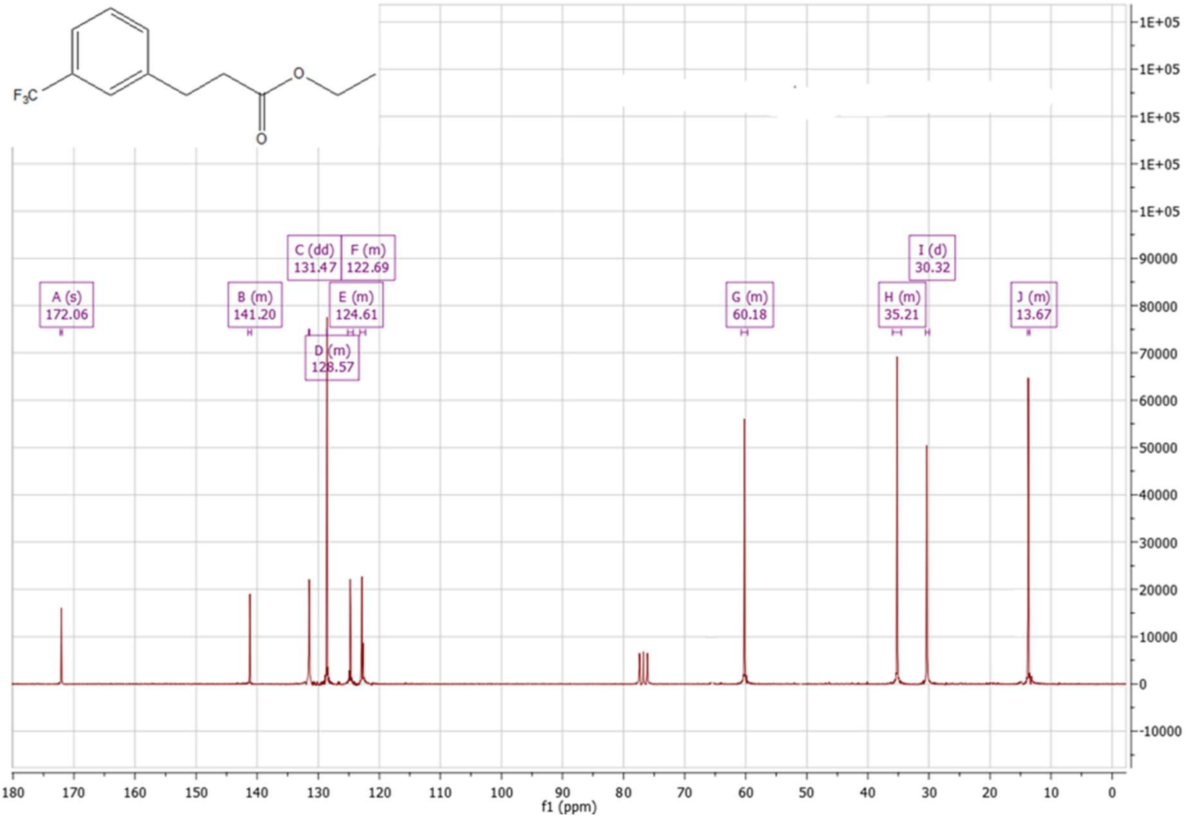

Fig S4. ^{13}C-NMR of Ethyl 3-(3-(trifluoromethyl)phenyl)propanoate (IV) (50 MHz, $CDCl_3$) : 172.06, 141.20, 131.47, 128.57, 124.61, 122.69, 60.18, 35.21, 13.69.

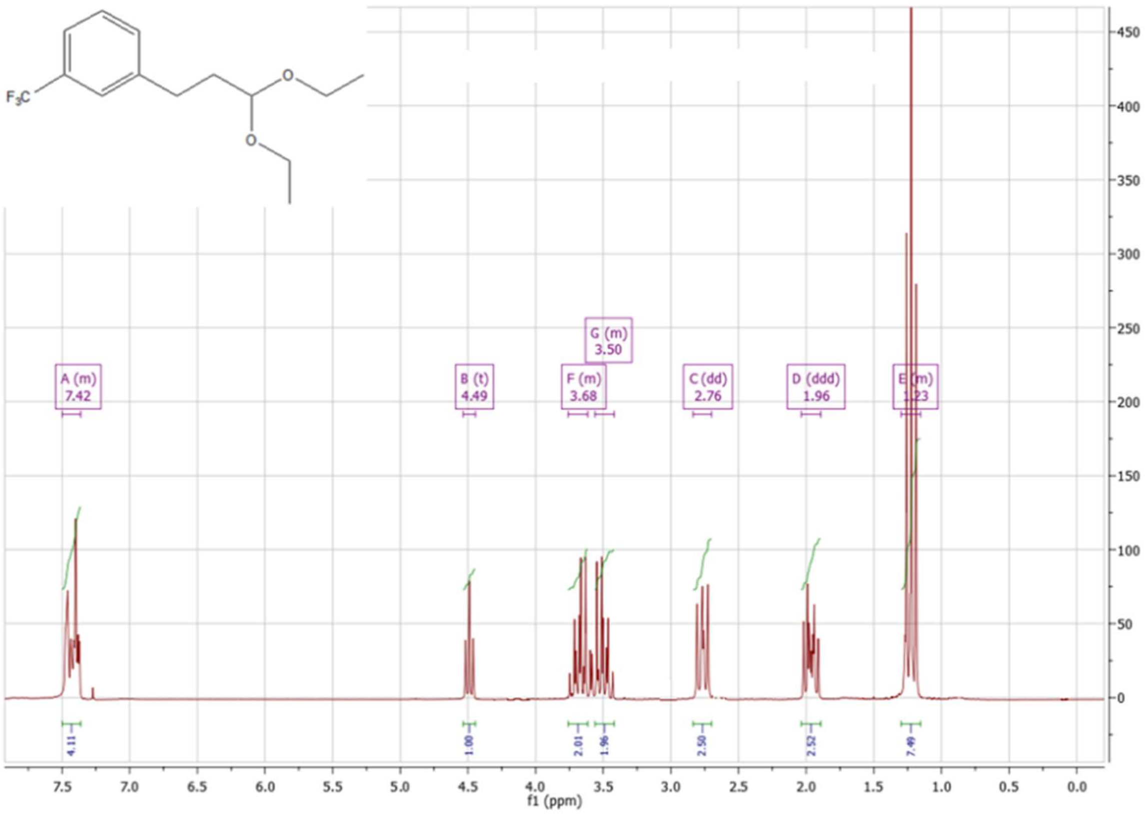

Fig S5. ^{1}H-NMR of 1-(3,3-Diethoxypropyl)-3-(trifluoromethyl)benzene (III) (300MHz, $CDCl_3$): 7.47-7.37 (m, 4H), 4.52-4.46 (t, J = 5.7, 9.9 Hz, 1H), 3.75-3.63 (q, J = 7.2, 9.6 Hz, 2H), 3.54-3.43 (q, J = 6.9, 9.3 Hz, 2H), 2.81-2.73 (q, J = 8.1, 10.2 Hz, 2H), 2.02-1.91 (q, J = 8.1, 10.1 Hz, 2H), 1.26-1.22 (t, J = 7.2, 11.4 Hz, 6H).

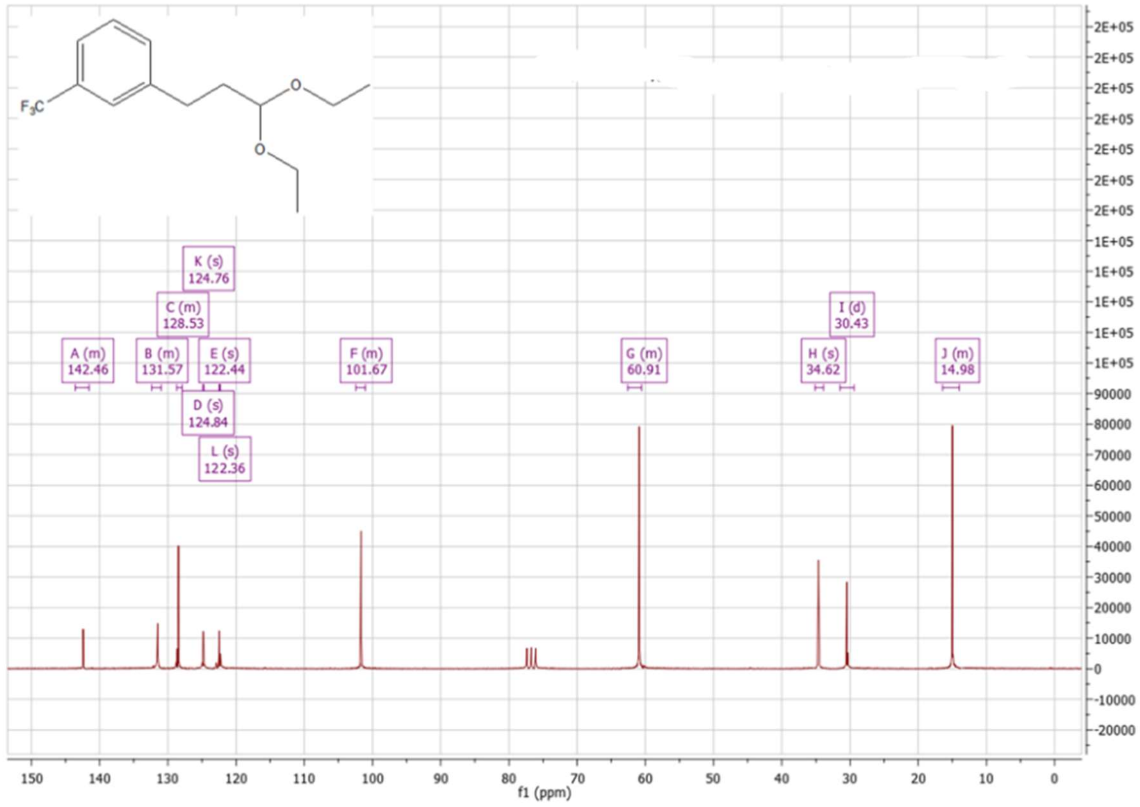

Fig S6. ^{13}C-NMR of 1-(3,3-Diethoxypropyl)-3-(trifluoromethyl)benzene (III) (50 MHz, $CDCl_3$): 142.46, 131.57, 128.53, 124.84, 124.76, 122.44, 122.36, 101.67, 60.91, 34.62, 30.43, 14.98.

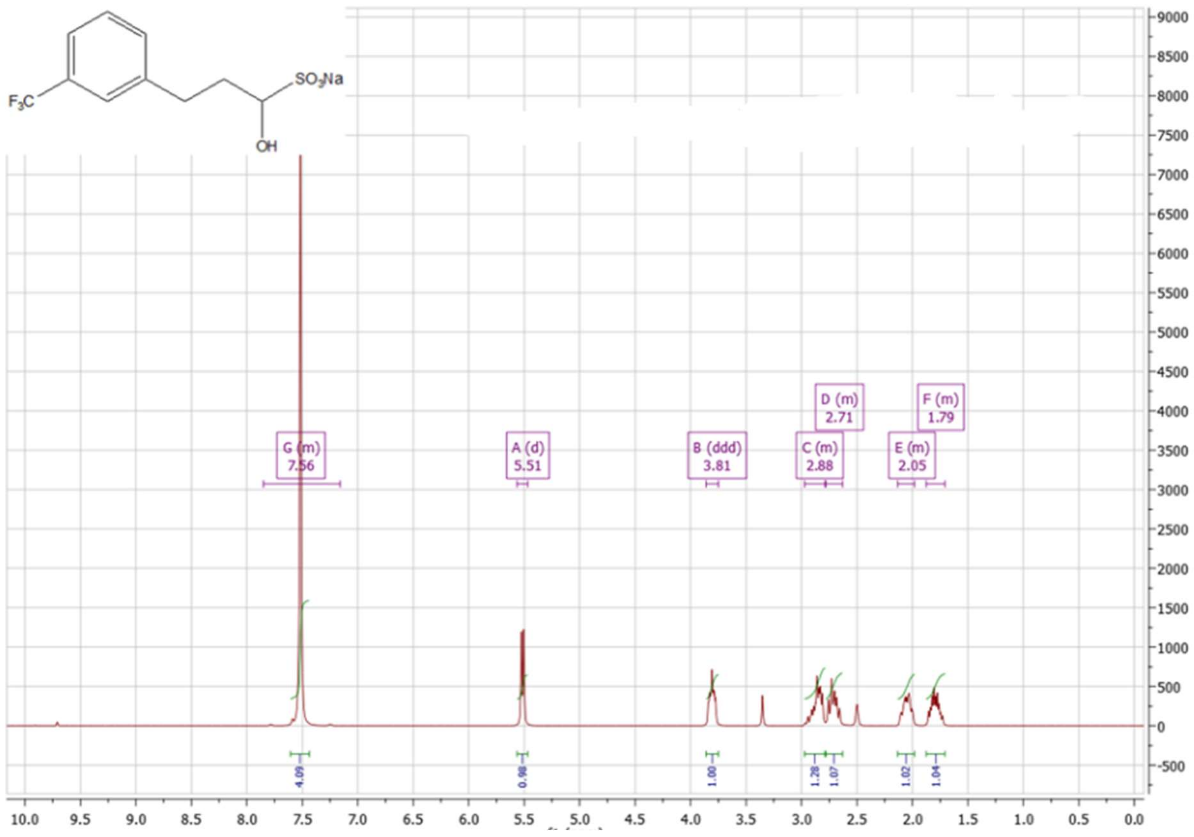

Fig S7. ^{1}H-NMR of Sodium 1-hydroxy-3-(3-trifluoromethylphenyl)propane-1-sulfonate (300 MHz, DMSO-d_6): 7.81-7.25 (m, 4H), 5.52-5.50 (d, J = 6 Hz, 1H), 3.84-3.78 (m, 1H), 2.94-2.81 (m, 1H), 2.76-2.65 (m, 1H), 2.11-2.00 (m, 1H), 1.85-1.73 (m, 1H).

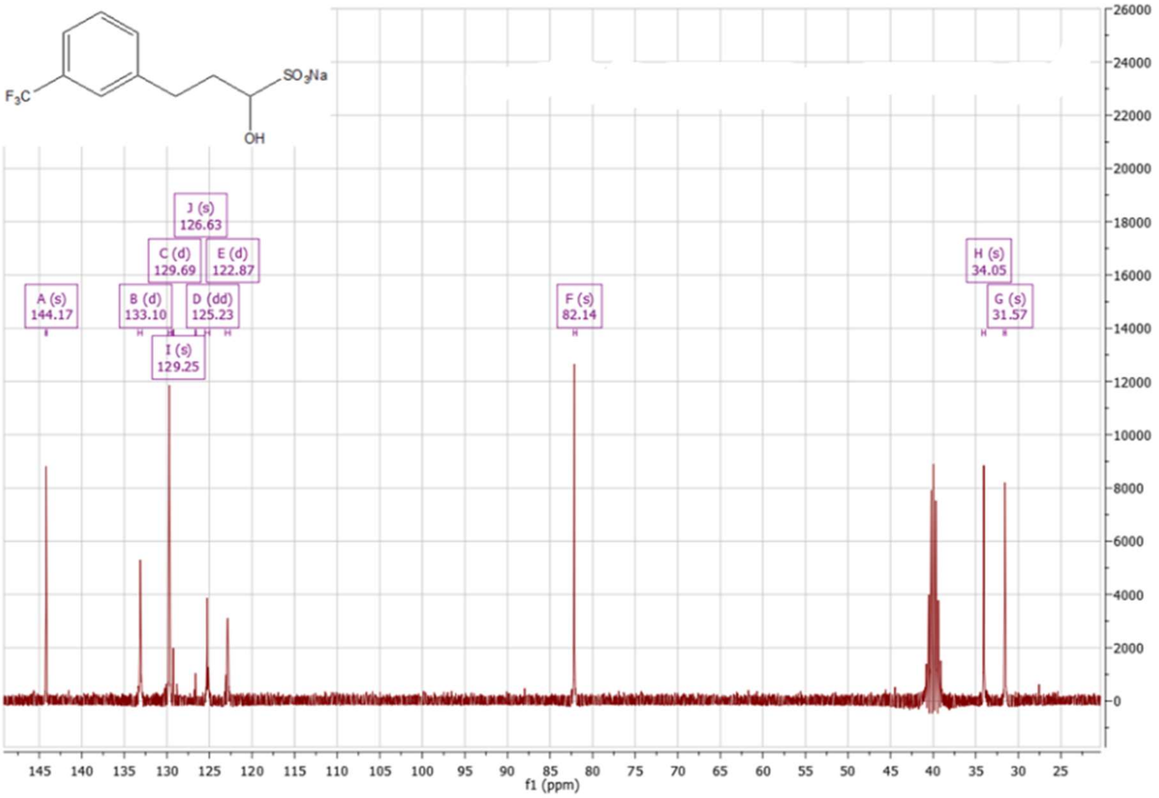

Fig S8. ^{13}C-NMR of Sodium 1-hydroxy-3-(3-trifluoromethylphenyl)propane-1-sulfonate (76 MHz, DMSO-d_6) : 144.17, 133.10, 129.69, 129.25, 126.63, 125.23, 122.87, 82.14, 34.05, 31.57.

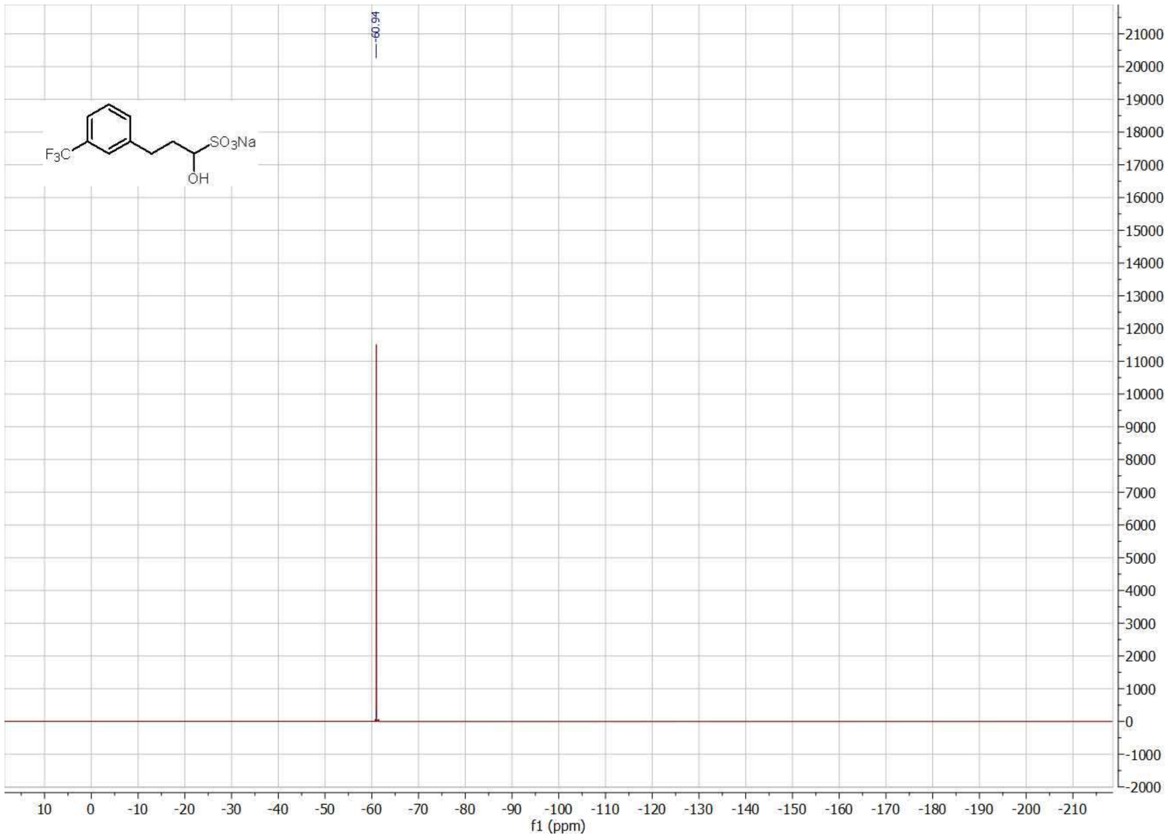

Fig S9. ^{19}F NMR of Sodium 1-hydroxy-3-(3-trifluoromethylphenyl)propane-1-sulfonate

(400 MHz, DMSO-d_6) : -60,9

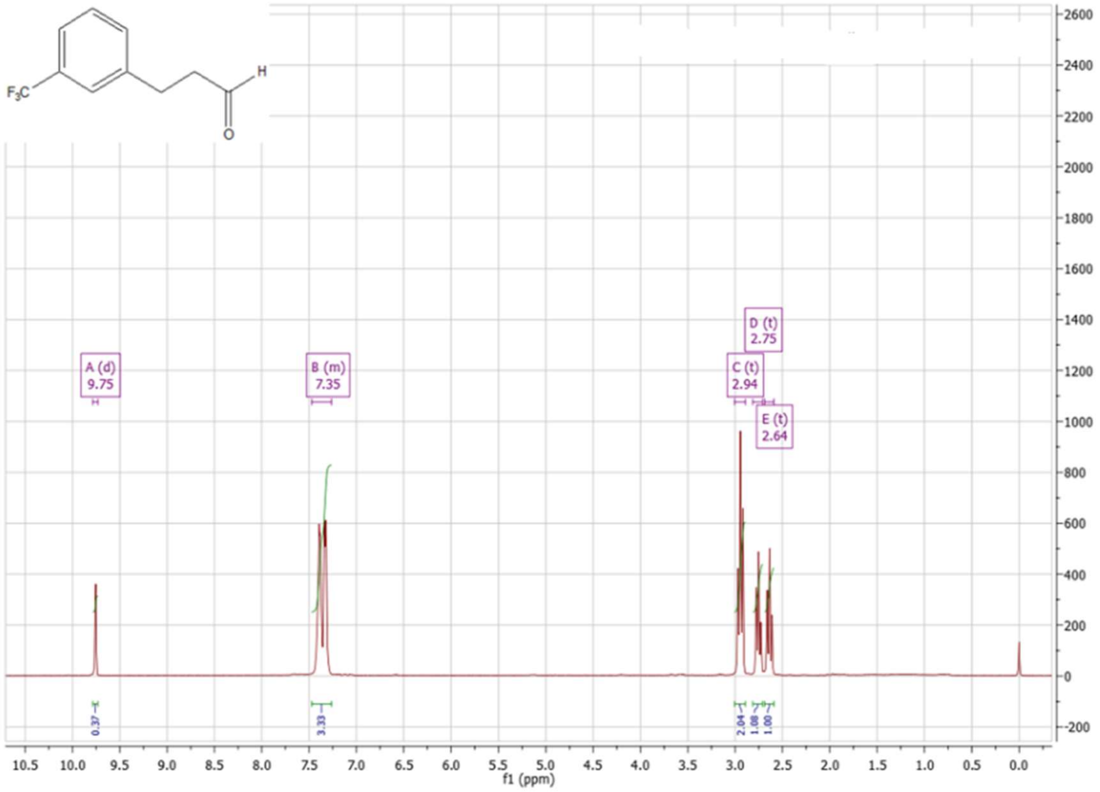

Fig S10. ^{1}H-NMR of 3-(3-(Trifluoromethyl)phenyl)propanal (**II**) (300MHz, $CDCl_3$): 9.75 (s, 1H), 7.38-7.31 (m, 4H), 2.95-2.91 (t, *J* = 7.5, 15 Hz, 2H), 2.77-2.73 (t, *J* = 7.8, 14.9 Hz, 1H), 2.66-2.62 (t, J = 7.5, 15, 1H).

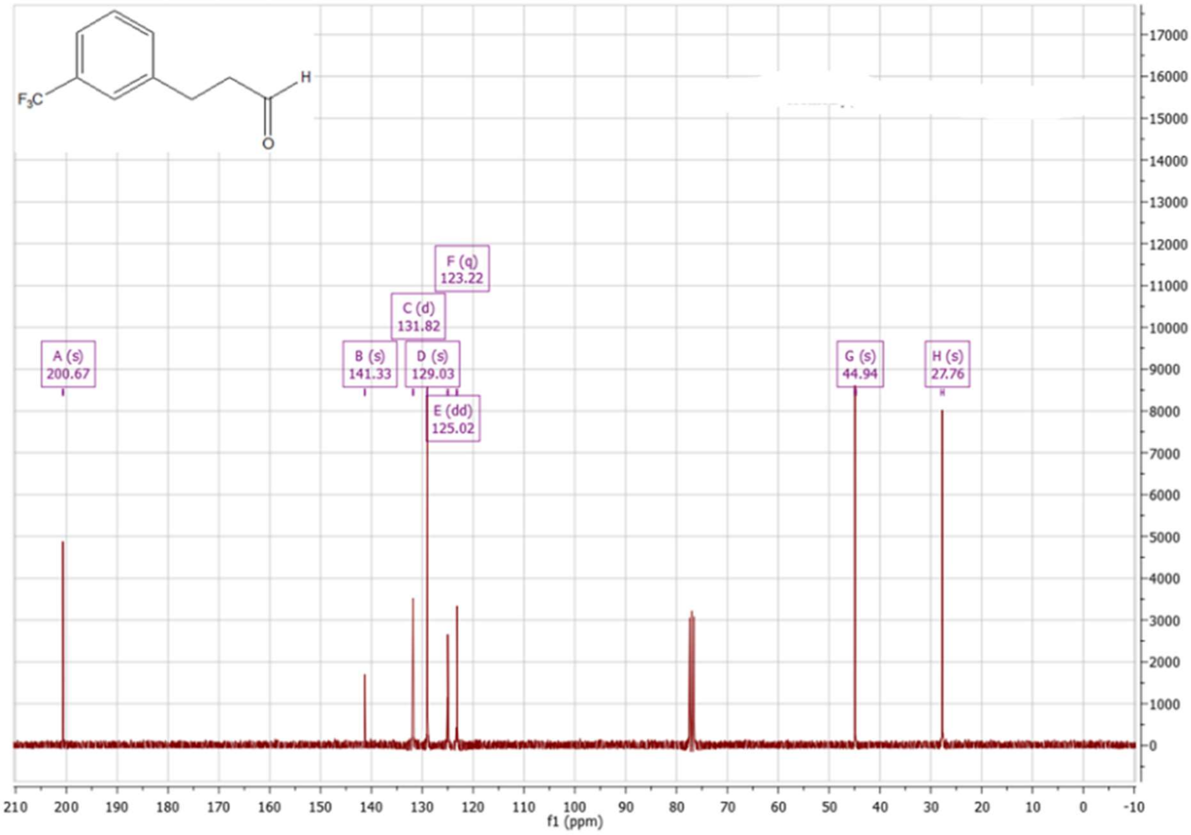

Fig S11. ^{13}C-NMR of 3-(3-(Trifluoromethyl)phenyl)propanal (II) (76 MHz, $CDCl_3$): 200.67, 141.33, 131.82, 129.03, 125.02, 123.22, 44.94, 27.76.

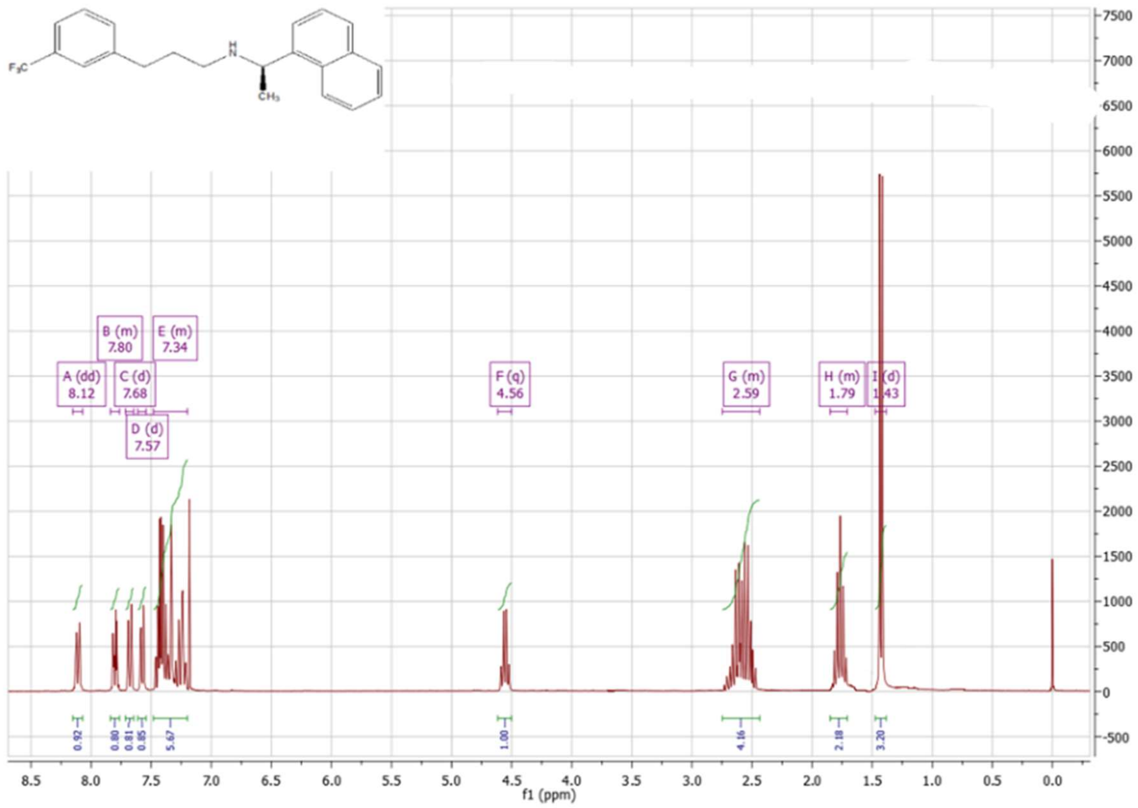

Fig S12. ^{1}H-NMR of 3-(3-(Trifluoromethyl)phenyl)-*N*-((*R*)-1-(naphthalen-1-yl)ethyl)propan-1-amine (I) (300MHz, $CDCl_3$): 8.13-8.11 (d, J = 9.3 Hz 1H), 7.82-7.78 (dd, J = 7.2, 8.1 Hz, 1H), 7.69-7.67 (d, J = 8.1 Hz, 1H), 7.58-7.56 (d, J = 6.3 Hz, 1H), 7.44-7.24 (m, 6H), 4.58-4.51 (q, J = 6.6 Hz, 1H), 2.75-2.49 (m, 4H), 1.81-1.72 (m, 2H), 1.44-1.42 (d, J = 6.6 Hz, 3H).

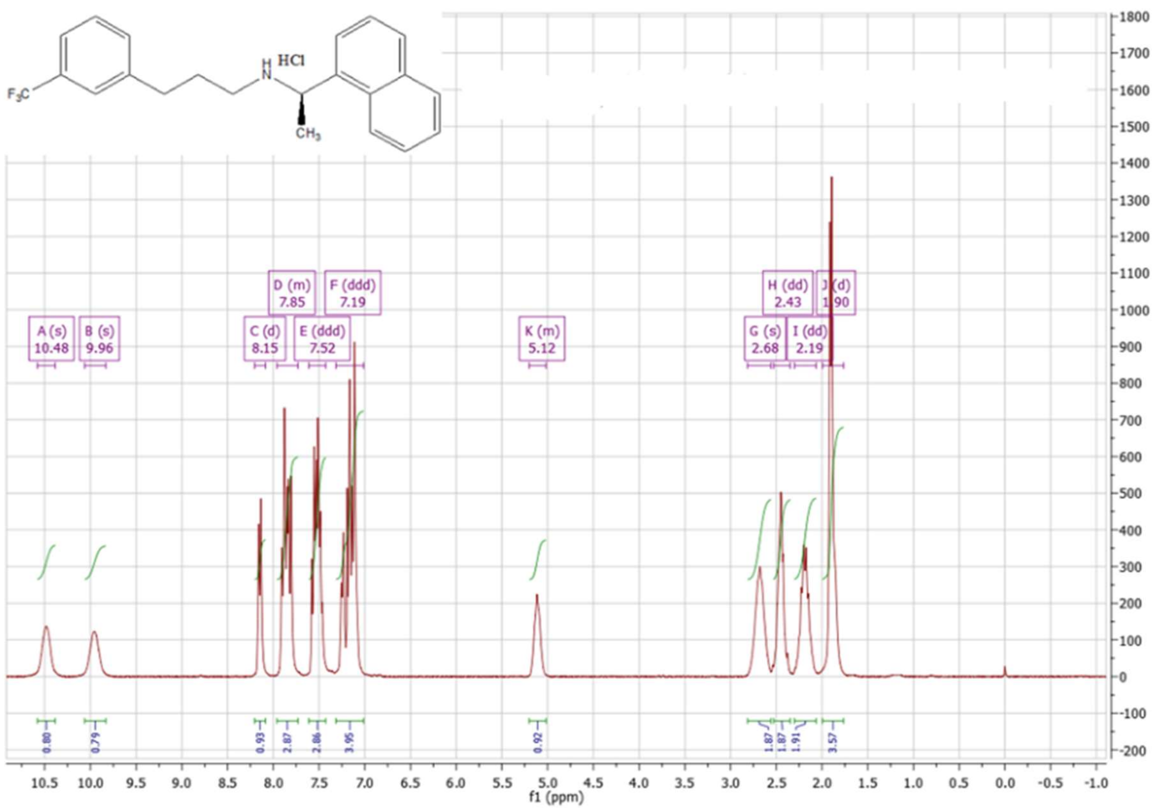

Fig S13. ¹H-NMR of 3-(3-(trifluoromethyl)phenyl)-*N*-((*R*)-1-(naphthalen-1-yl)ethyl)propan-1-amine hydrochloride (300 MHz, $CDCl_3$): 10.48 (broad s, 1H), 9.96 (broad s, 1H), 8.16-8.14 (d, J = 9 Hz, 1H), 7.90-7.80 (m, 3H), 7.57-7.46 (m, 3H), 7.26-7.12 (m, 4H), 5.16-5.08 (m, 1H), 2.72-2.64 (m, 2H), 2.46-2.39 (m, 2H), 2.23-2.15 (m, 2H), 1.94-1.86 (d, J = 6 Hz 1H).

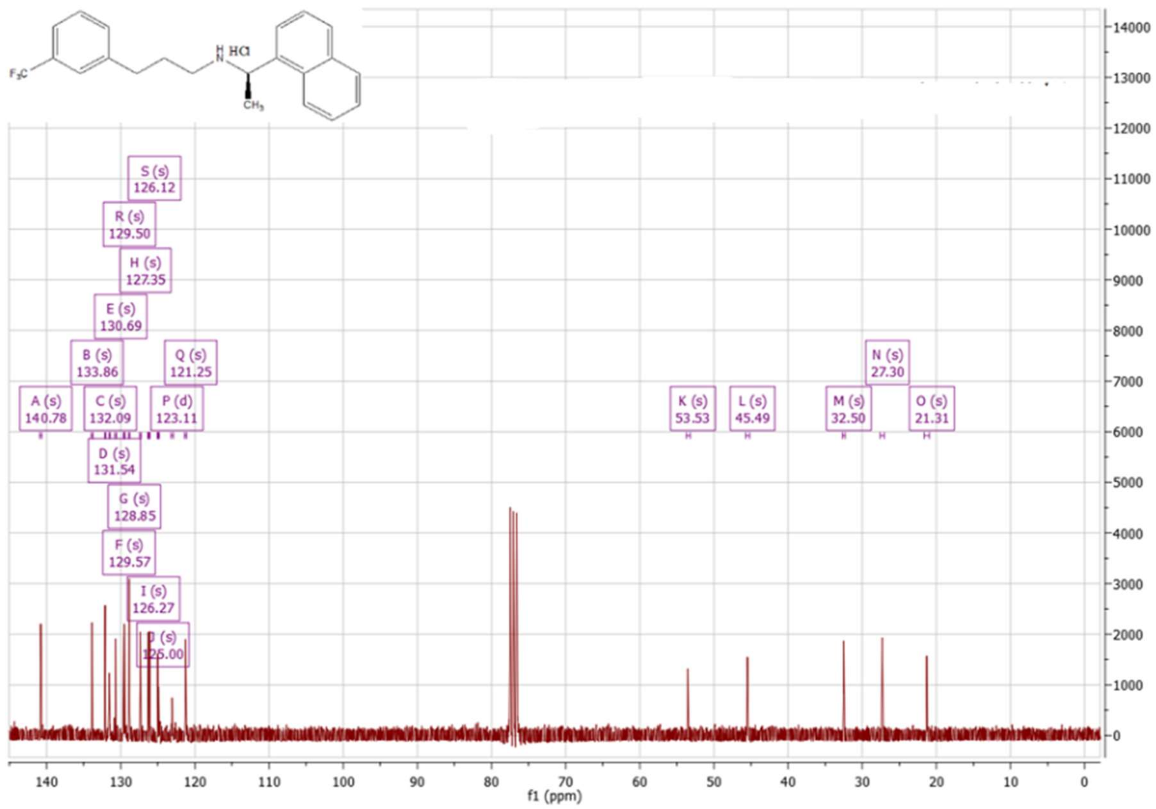

Fig S14. ^{13}C-NMR of 3-(3-(trifluoromethyl)phenyl)-*N*-((*R*)-1-(naphthalen-1-yl)ethyl)propan-1-amine hydrochloride (76 MHz, $CDCl_3$): 140.78, 133.86, 132.09, 131.54, 130.69, 129.57, 129.50, 128.85, 127.35, 126.27, 126.12, 125.00, 124.90, 123.1, 121.25, 53.53, 45.49, 32.50, 27.30, 21.31.

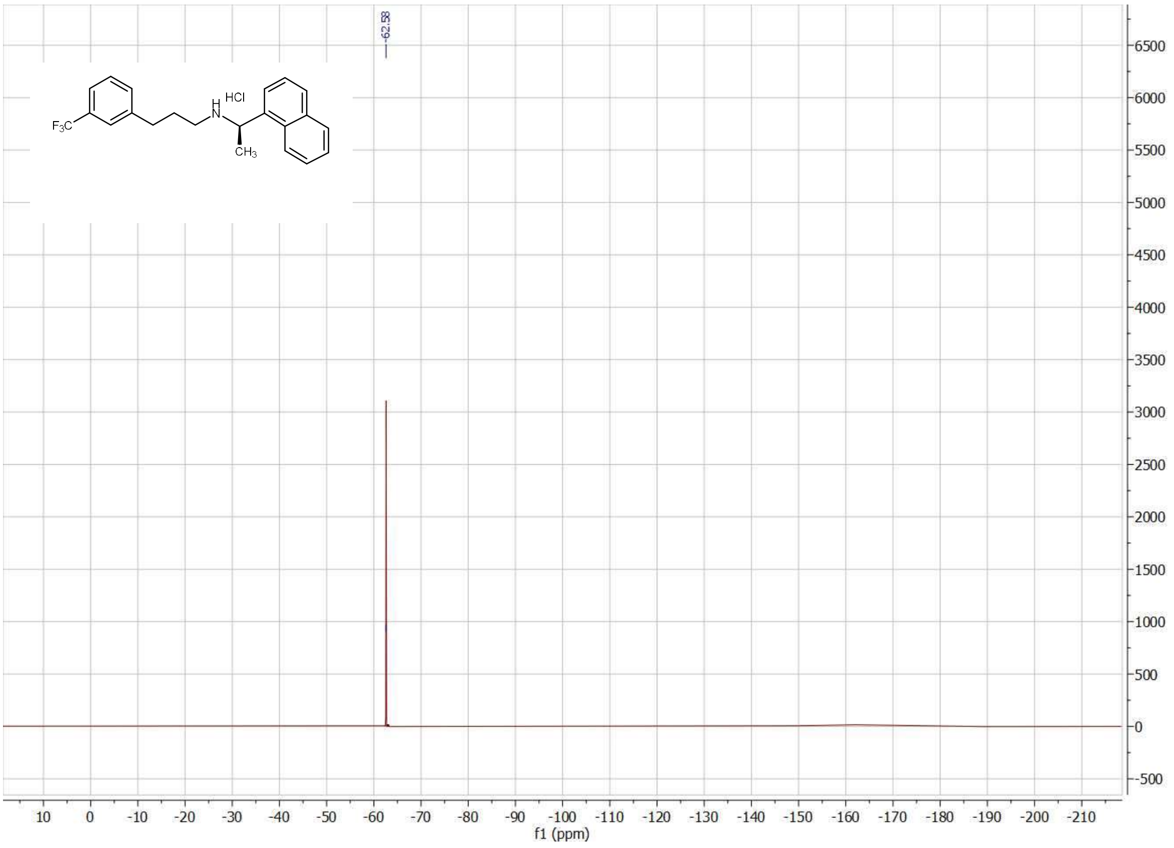

Fig S15. ^{19}F-NMR of 3-(3-(trifluoromethyl)phenyl)-*N*-((*R*)-1-(naphthalen-1-yl)ethyl)propan-1-amine hydrochloride (400 MHz, $CDCl_3$): -62,6
